# Automated Segmentation of Mass Lesions in Mammography: Mask R-CNN Versus Classical Methods, Fuzzy Clustering and MedSAM

**DOI:** 10.3390/diagnostics16203289

**Published:** 2026-10-10

**Authors:** Marta Vicentini, Martina Oria, Eva Raina, Manuela Durando, Alessandra Manzin

**Affiliations:** 1 Department of Advanced Materials Metrology and Life Sciences, Istituto Nazionale di Ricerca Metrologica (INRiM), 10135 Torino, Italy; martina.oria@polito.it (M.O.); eva.raina@polito.it (E.R.); 2 Department of Electronics and Telecommunications, Politecnico di Torino, 10129 Torino, Italy; 3 Department of Diagnostic Imaging and Interventional Radiology, Azienda Ospedaliera Universitaria Città della Salute e della Scienza di Torino, 10126 Torino, Italy; manuela.durando@unito.it

**Keywords:** breast cancer screening, digital mammography, computer-aided diagnosis, medical image segmentation, mass lesion annotation, convolutional neural networks, classical segmentation methods, performance evaluation metrics, radiologist assessment

## Abstract

**Background/Objectives:** The automatic and accurate segmentation of mass lesions in mammograms is a critical step in the computer-aided diagnosis workflow. This study evaluates the mask region-based convolutional neural network (Mask R-CNN), a robust deep learning model for instance-level segmentation, highlighting its growing potential in medical imaging. **Methods:** Following configuration optimization, the model is applied to mammography images from the CSAW-CC and TOMPEI-CMMDs, which provide masks annotated by breast radiology experts, to be used as a ground truth. To quantify shape and contour discrepancies with respect to the radiologists’ annotations, performance is evaluated with both overlap- and distance-based metrics. Furthermore, the model is tested on synthetic mammograms and benchmarked against classical segmentation methods (Otsu thresholding, gradient vector flow active contour, and Chan–Vese segmentation), Fuzzy c-means clustering, and the state-of-the-art Medical Segment Anything (MedSAM) model. **Results:** By leveraging transfer learning, a limited dataset suffices for stable training, with the network converging by the 10th epoch. The optimal configuration achieves a bounding box average precision (BBox AP) of 0.80 and a segmentation average precision (Segm AP) of 0.58. Evaluated on a test set of real mammograms, the model correctly identifies the lesions, yielding an average Dice similarity coefficient (DSC) of 0.87 and an average intersection over union (IoU) of 0.77, performance aligned with the current state of the art. **Conclusions:** Mask R-CNN has emerged as a reliable and robust model for breast lesion segmentation; compared to the other methods evaluated in this study, it demonstrates superior performance across both overlap- and distance-based metrics. The model exhibits high DSC and IoU scores even on synthetic mammograms with a priori known lesion profiles, which can be used as an objective ground truth to mitigate annotation bias from inter-operator variability among radiologists.

## 1. Introduction

Automatic lesion segmentation is a critical prerequisite for modern computer-aided diagnosis (CAD) systems in breast cancer screening with mammography [1,2,3,4]. Isolating a suspected tumor from the surrounding healthy tissue allows automated algorithms to extract tumor size and shape features, classify pathologies, and track changes over time. Segmentation algorithms can provide reproducible boundaries, eliminating subjectivity and inter-observer variability related to manual delineation, and can enable high-throughput processing and large screening workflows.

Among breast abnormalities, masses are particularly difficult to analyze, due to the high variability in shape, size, and margin definition; spiculated margins often blend seamlessly into surrounding parenchymal tissue. Early CAD systems relied on classical approaches, like global/local thresholding, iterative pixel classification, edge detection, template matching and stochastic relaxation [5]; however, these techniques often fail when handling diverse tissue textures and complex mass margins. Active contour models, able to dynamically adapt to local intensity variations, have also been proposed to reduce interference from surrounding tissues and allow more precise contouring of irregular lesion boundaries [6].

Recently, convolutional neural networks (CNNs) have been investigated to automate feature extraction directly from pixel data, and specialized architectures, like the U-shaped network (U-Net) [7,8,9,10,11] and novel structures based on residual networks (ResNet) [12], have led to a significant improvement in segmentation accuracy. Improvements have also been observed by combining CNNs with classical approaches, such as Otsu thresholding plus morphological operations [13], and active contour models [14]. Despite these gains, existing automated techniques still face major challenges; fibroglandular or dense tissue can mask underlying masses, highly irregular shapes can lead to under-segmentation, and issues can arise when capturing localized margin details and the present global structural context. Moreover, the models should guarantee generalizability across various mammography hardware vendors without severe performance drops, considering that different post-processing software can lead to images with strong variations in sharpness, contrast, gray level distribution, and signal-to-noise ratio [15].

Another critical aspect is the need for large-scale databases with radiological annotations. In the field of digital mammography, there are several databases built collecting images from breast cancer screening, like the Digital Database for Screening Mammography (DDSM) [16], INbreast [17], the Swedish Cohort of Screen-Age Women (CSAW) [18], the Vietnamese dataset of digital mammography (VinDr-Mammo) [19], the Chinese Mammography Database (CMMD) [20], the US EMory BrEast imaging Dataset (EMBED) [21], and the UK OPTIMAM Medical Image Database (OMI-DB) [22]. Among these, only the Curated Breast Imaging Subset (CBIS) of DDSM [23], INbreast, and the updated versions of CSAW and CMMD, namely CSAW-CC [24] and TOMPEI-CMMD [25], contain annotated masks with precise mass boundaries. These can be exploited for the training of CNNs for segmentation tasks and used as a ground truth for method benchmarking, whereas VinDr-Mammo, EMBED and OMI-DB include rectangular bounding boxes around potential cancerous areas. It is also worth noting that the period over which the stored images were acquired can vary significantly from one database to another. Although CBIS-DDSM and INbreast are among the most widely used databases for comparing segmentation CNNs [17,26,27,28,29,30,31,32,33,34,35], these include mammograms acquired over 15 years ago (collected in the periods of 1993–1999 and 2008–2010, respectively), which is a factor that severely limits the ability of the models to generalize to the latest acquisition systems. Moreover, CBIS-DDSM does not contain digital mammograms, but scans of analog films.

In this framework, we analyze the performance of the mask region-based convolutional neural network (Mask R-CNN) [36] when applied to the segmentation of mass lesions in digital mammograms from the CSAW-CC [24], TOMPEI-CMMD [25,37], and INbreast [17] databases. Mask R-CNN is an advanced deep learning model for instance segmentation, recently used in medical imaging to obtain precise pixel-level boundaries of anatomical structures or pathologies. High accuracy has been achieved for magnetic resonance imaging (MRI) and computed tomography (CT) images [38,39,40,41,42,43,44,45], and some studies have shown its great potential also in the field of mammography, with applications from two-dimensional (2D) to three-dimensional (3D) imaging [46,47,48,49,50].

First, we evaluate Mask R-CNN within the training and validation pipeline to determine the optimal model configuration relative to the minimal baseline, yielding the best object detection and instance segmentation performance. As a methodological contribution for testing the optimal configuration identified, we use synthetic mammograms with artificially inserted lesions as an evaluation benchmark, ensuring a perfectly controlled ground truth. Subsequently, we conduct an extended evaluation of model performance on patches extracted from real mammograms, focusing on the impact of data source, vendor style, as well as lesion size and morphology. The superior performance of Mask R-CNN is highlighted in comparison with several segmentation models, including classical segmentation methods such as Otsu thresholding [51], the gradient vector flow (GVF) active contour method [52], and Chan–Vese segmentation (CVS) [53], in addition to a clustering algorithm like Fuzzy c-means (FCM) [54], and Meta’s state-of-the-art (SOTA) model, Medical Segment Anything (MedSAM) [55]. The benchmarking is performed considering not only overlap-based metrics, typically used for evaluating segmentation models, but also distance-based metrics, as a tool for quantifying the spatial proximity and anatomical accuracy of the predicted boundaries.

## 2. Materials and Methods

### 2.1. Data Source

As data sources for training and validation of Mask R-CNN, we consider the open-access databases CSAW-CC [18,24] and TOMPEI-CMMD [20,25,37], which have the advantage of providing mask annotations, generated with a rigorous segmentation pipeline involving breast radiology experts. The total number of images containing annotated masses selected from CSAW-CC and TOMPEI-CMMD is 188 and 184, respectively. The training set comprises 150 images from CSAW-CC and 145 from TOMPEI-CMMD, while the validation set contains 38 images from CSAW-CC and 38 images from TOMPEI-CMMD. Considering the two databases together enables us to include mammograms from different vendors, as the CSAW images were acquired with Hologic scanners, while the TOMPEI-CMMD ones were acquired with GE HealthCare scanners.

To target the segmentation model towards a precise delineation of lesion contours, the training/validation dataset is built by extracting rectangular patches centered on masses and expanded by 25 px with respect to the minimum and maximum boundaries along horizontal and vertical directions. From CSAW-CC, whose images have a spatial resolution of 70 μm and a bit depth of 14, we obtain patches with an average size of 52,955 px (min: 800 px, max: 484,250 px), corresponding to an average mass area of 32,235 px (min: 492 px, max: 315,692 px). From TOMPEI-CMMD, whose images have a spatial resolution of 94 μm and a bit depth of 8, we obtain patches with an average size of 124,144 px (min: 4556 px, max: 838,537 px), corresponding to an average mass area of 70,829 px (min: 2994 px, max: 512,803 px). The used images are in Digital Imaging and COmmunications in Medicine (DICOM) format, in vendor-processed or “for presentation” style. All extracted patches are converted to 8-bit grayscale and then normalized to pixel values between 0 and 1 using min–max linear normalization.

For the test phase, in addition to CSAW-CC and TOMPEI-CMMD, we consider the INbreast database, which includes mammography images with annotations generated with the software OsiriX (Pixmeo SARL, Geneva, Switzerland) [56] and rigorously validated by specialist radiologists. INbreast is used to evaluate the model’s ability to segment images with a vendor style not seen in the training, acquired with Siemens scanners. Moreover, it serves as a basis for comparison with other segmentation networks typically benchmarked on INbreast.

Details regarding each mammogram from which we extract patches for the experimental analysis reported in Section 3.2, Section 3.3 and Section 4 are provided in Table S8 of the Supplementary Materials. These include the image label, laterality, view, lesion information, and bounding box coordinates.

### 2.2. Instance Segmentation Framework

**Figure 1:**
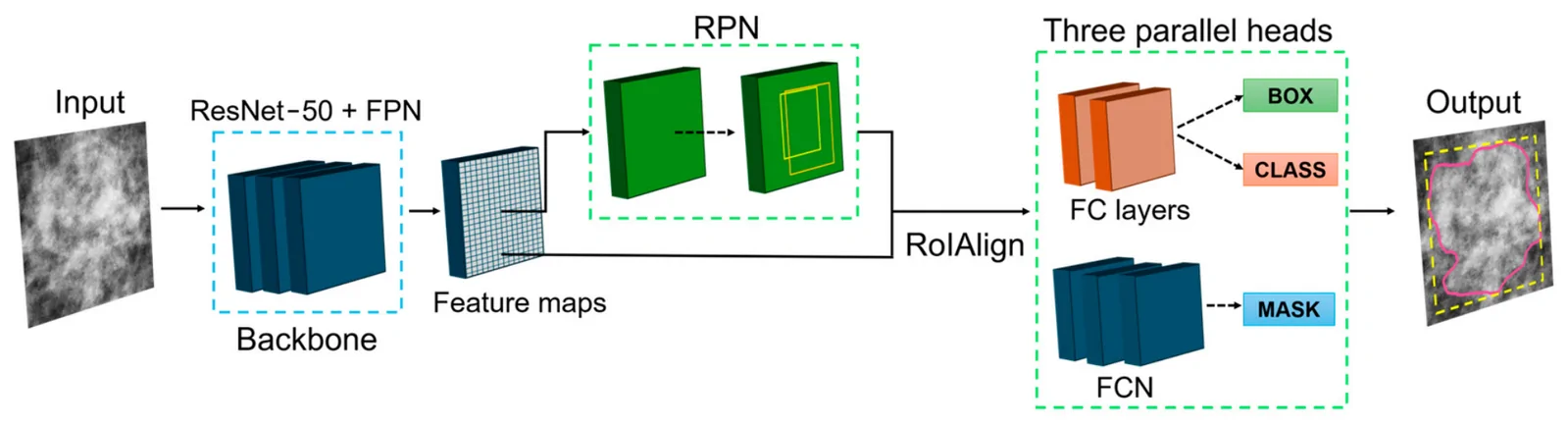
Schematics of the deep learning segmentation model based on Mask R-CNN used for the contouring of breast lesion masses.

We apply an instance segmentation framework based on Mask R-CNN, a well-known deep learning segmentation model developed as an extension of the faster region-based convolutional neural network (Faster R-CNN) for object detection [57], by introducing a segmentation branch dedicated to mask prediction [36]. As schematized in Figure 1, the initial stages of the model coincide with those of the Faster R-CNN architecture: multiscale feature maps are generated from a backbone network (here ResNet-50), combined with a feature pyramid network (FPN) for object detection at different scales [58].

The availability of information at different resolutions is particularly relevant in the context of mammography, where masses can exhibit high variability in terms of size, morphology, and contrast. Subsequently, a region proposal network (RPN) processes the feature maps to generate a set of possible bounding boxes. These are further refined in a second stage using the region of interest alignment (RoI Align) module, which is one of the distinctive elements of Mask R-CNN. Unlike the traditional RoI Pooling integrated in the Faster R-CNN model, RoI Align eliminates quantization errors through bilinear interpolation, preserving accurate spatial alignment of features: a crucial property for the correct delineation of mass contours. Finally, for each RoI, the model implements three parallel heads, each dedicated to a specific task: (i) a regression head, which flattens the feature map through fully connected (FC) layers to optimize the spatial coordinates of the bounding box for more precise mass localization; (ii) a classification head, which utilizes FC layers to make a binary prediction (mass vs. background), determining the presence or absence of a breast mass; and (iii) a segmentation head, implemented as a fully convolutional network (FCN) to preserve spatial topology, which produces a pixel-wise binary mask for an accurate representation of the mass shape.

In this study, we utilize the “torchvision” implementation of Mask R-CNN in PyTorch 2.11.0, applying a transfer learning approach with weights pre-trained on the ImageNet dataset [59]. This strategy enables the exploitation of general visual features learned from large-scale natural images, guaranteeing an improvement in convergence speed and overall performance also in the specific domain of mammography.

### 2.3. Determination of Optimal Configuration

To determine the optimal model configuration along the training/validation pipeline, we conduct an optimization study by adding a custom optimizer, a learning rate (LR) scheduler and data augmentation techniques to a minimal baseline configuration. Four model configurations are defined and compared. The baseline configuration (Model 1) employs the stochastic gradient descent (SGD) optimization algorithm with default hyperparameters and without any LR scheduling strategy. The second configuration (Model 2) introduces a customized SGD optimizer with an LR of 0.005, a momentum of 0.9, and a weight decay of 5 × 10^−4^. The third configuration (Model 3) further extends Model 2 by incorporating a StepLR scheduler, which decays the LR by a multiplicative factor γ = 0.1 every 5 epochs. Finally, Model 3 is integrated with a data augmentation pipeline (Model 4) including horizontal and vertical flips (applied with probability *p* = 0.5), random 90° rotations (*p* = 0.5), brightness and contrast jittering (*p* = 0.5), Gaussian blur (*p* = 0.3), Gaussian noise injection (*p* = 0.3), and elastic deformations (*p* = 0.3). All models are trained up to the 25th epoch; to ensure full reproducibility of the experiments, the random seed is fixed and consistently applied across all stochastic components involved in training. All hyperparameters used for Model 3 are listed in Table S1 of the Supplementary Materials.

**Figure 2:**
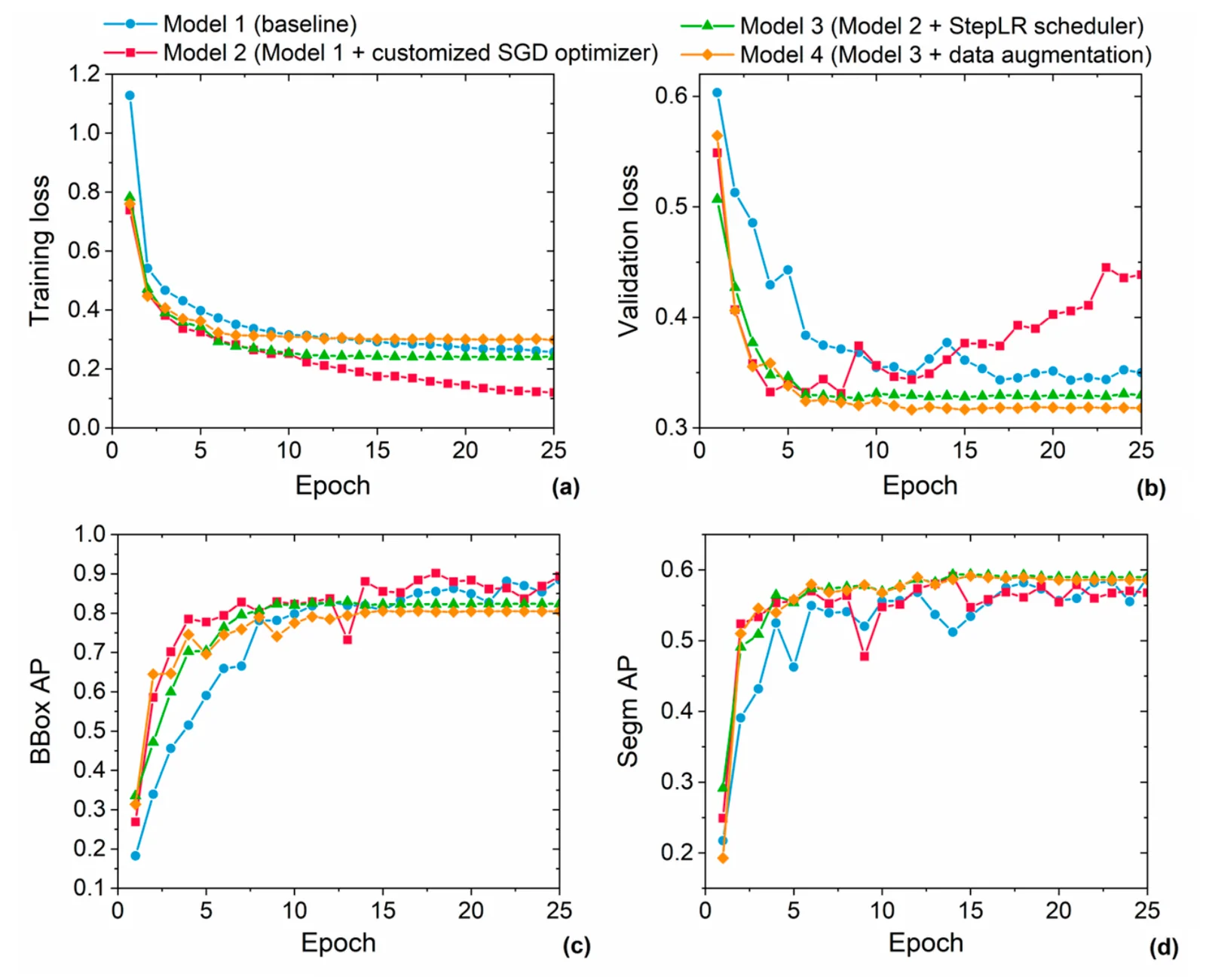
Comparison of Mask R-CNN model configurations along the training/validation pipeline, in terms of (**a**) training loss, (**b**) validation loss, (**c**) bounding box average precision (BBox AP) and (**d**) segmentation average precision (Segm AP). For BBox AP and Segm AP values, we consider IoU = 0.5:0.95.

Each model is evaluated using training and validation losses, alongside common objects in context (COCO) metrics for object detection and instance segmentation, including bounding box average precision (BBox AP) and segmentation average precision (Segm AP) averaged over intersection over union (IoU), with thresholds from 0.50 to 0.95 [60,61]. As shown in Figure 2, all configurations exhibit smooth convergence in terms of total training loss. However, Model 2 presents a marked increase in validation loss after the 12th epoch, as an indication of overfitting. The introduction of the StepLR scheduler in Model 3 stabilizes the validation loss, confirming its regularizing effect. Model 4 achieves only a slightly lower validation loss, but no significant improvements are observed for the BBox AP and Segm AP metrics, indicating that data augmentation does not provide an important benefit in this configuration, likely due to the already strong generalization ability of the pre-trained backbone. Based on this analysis, Model 3 is selected as the optimal configuration, as it offers the best trade-off between validation stability, segmentation performance and computational cost. Moreover, retraining Model 3 using two other random seeds produces similar loss results, as reported in Figure S1 of the Supplementary Materials, confirming the stability of the selected configuration.

**Figure 3:**
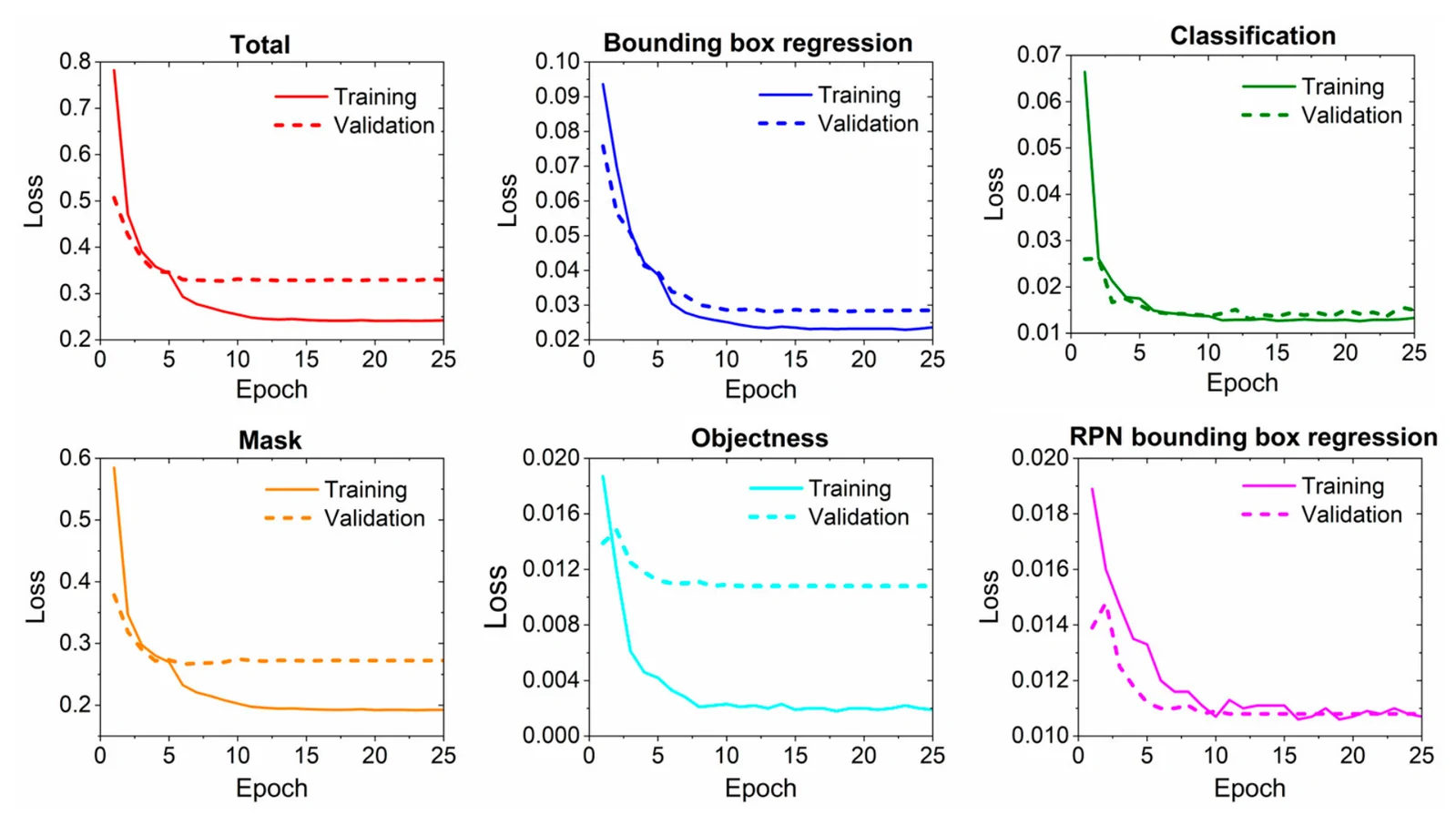
Loss components vs. epoch of the Mask R-CNN model configuration with StepLR scheduler (Model 3), during training and validation phases. The panels contain (from left to right, top to bottom) the total, bounding box regression, classification, mask, objectness and RPN bounding box regression losses. The solid lines represent the training curves, whereas the dotted lines indicate the corresponding validation curves.

Figure 3 illustrates the trend, during training and validation phases, of the individual loss components of Model 3, namely bounding box regression loss, classification loss, mask loss, objectness loss, and RPN bounding box regression loss. All contributions practically stabilize after the 10th epoch, with mask loss being the dominant component, with values above 0.2 during the first 10 epochs of training and always higher than 0.25 for the validation phase.

Regarding computational time for Model 3, the training and validation phase for 372 images over 25 epochs takes approximately 15 min on an NVIDIA A100 (80 GB) graphics processing unit (GPU). On the same GPU, the average inference time is 0.18 s, compared to 3.81 s on a 32-core central processing unit (CPU) with 32 GB of random-access memory (RAM).

### 2.4. Segmentation Methods for Comparison

The Mask R-CNN model is compared to classical segmentation methods, including Otsu thresholding, gradient vector flow (GVF) active contour method, and Chan–Vese segmentation (CVS), as well as the Fuzzy c-means (FCM) clustering algorithm, and a SOTA deep learning model for medical image segmentation, MedSAM.

Otsu thresholding is a method that has been the benchmark for early segmentation techniques, since it is an effective and fast automatic global method, easy to implement [51]. It analyses pixel intensity distribution to search for an optimal cut-off to binarize the image, resulting in a final black-and-white image where white shapes represent objects of interest. GVF and CVS are based on active contours (snakes), where an initial curve iteratively evolves toward the boundaries of the target object. A snake is an active contour model defined as a deformable spline that minimizes an energy functional, under the influence of external constraint forces and internal image features like texture and gradients, which guide the curve evolution. The GVF active contour method, which was originally applied in medical imaging, is an edge-based segmentation technique that extends the classical parametric snake model [52,62]. One of the key advantages of the GVF method lies in its ability to increase the snake flexibility and capture range, enabling the contour to accurately delineate objects of complex shapes, even in the presence of concavities [63,64]. Considering the parametric curve, *C*(*s*) = [*x*(*s*), *y*(*s*)], *s* ∈ [0, 1], which delineates the contour of an object within the greyscale image *I*, the dynamic equation governing the GVF snake is formulated as follows:

(1) $$\frac{\partial C}{\partial t}=\alpha \frac{\partial^{2}C}{\partial s^{2}}-\beta \frac{\partial^{4}C}{\partial s^{4}}+\kappa \nu$$

where α and β are parameters that control the snake tension and stiffness, respectively [52]. Parameter κ is introduced to balance the last term ν, ν(*s*) = [*u*(*s*), *v*(*s*)], which is the 2D GVF field determined by solving the equation:

(2) $$\mu \nabla^{2}\nu -\left(\nu -\nabla f\right){\left|\nabla f\right|}^{2}=0$$

where μ is a noise regularization parameter and $f={\left|\nabla g(I)\right|}^{2}$ is the edge map, with *g* being a processing function for image *I*.

On the other hand, in CVS, which is an iterative region-based method, the curve *C* is represented implicitly via the Lipschitz function φ, $C=\left\{\left(x,y\right)|\varphi \left(x,y\right)=0\right\}$, and its evolution is described by the zero-level curve at time *t* of the function $\varphi \left(t,x,y\right)$, whose dynamics are governed by

(3) $$\left\{\begin{array}{c} \frac{\partial \varphi }{\partial t}=h\left(\left|\nabla I\right|\right)\left|\nabla \varphi \right|\left[\nabla \cdot \left(\frac{\nabla \varphi }{\left|\nabla \varphi \right|}\right)\right]\\ \varphi \left(0,x,y\right)\;=\;\varphi_{0}\left(x,y\right) \end{array}\right.$$

where $\varphi_{0}\left(x,y\right)$ is the initial level set function and function *h* is defined as $h(\left|\nabla I\right|)={\left(1+{\left|\nabla G_{\sigma }I\right|}^{2}\right)}^{-1}$, with *G*_σ_ being a Gaussian filter with standard deviation σ. Through this approach, it is possible to split *C* into several curves when multiple objects are detected [53].

The FCM algorithm is an unsupervised clustering technique commonly employed in medical imaging for brain tissue segmentation and tumor detection in MRI scans [54,65,66,67]. The method, which processes the image as a one-dimensional array, is iterative and starts with a random assignment of membership degrees for each pixel relative to each of the *N* clusters. The partition, and consequently the cluster centers, are then updated by minimizing the following cost function:

(4) $$J={\sum_{i=1}^{P}\sum_{j=1}^{N}a_{ij}^{m}\left\|p_{i}-r_{j}\right\|}^{2}$$

where *P* is the total number of pixels, *a_ij_* is the degree of membership of pixel *p_i_* in the *j*-th cluster (with center *r_j_*), *m* is the degree of fuzziness of the partition, here fixed to 2, and $\left\|\;\cdot \;\right\|$ denotes the Euclidean norm [66]. In our application, we set the number of clusters *N* to 2, corresponding to the mass lesion and the surrounding tissue.

MedSAM is a foundation model obtained by fine-tuning the Segment Anything Model (SAM) on a medical dataset comprising 1,570,263 images, with their corresponding masks, covering 10 different imaging modalities and over 30 tumor types [55]. The predominant modalities are CT, MRI, and endoscopy, although the training dataset also includes ultrasound, fundus photography, dermatoscopy, and mammography images [55,68]. MedSAM has the same architectural structure as SAM, defined by three main components: an image encoder for processing and embedding images, a prompt encoder for converting prompts into embeddings, and a lightweight mask decoder for generating the final segmentation. Unlike the original SAM, MedSAM is designed to operate exclusively with bounding box prompts. In our application, MedSAM is run via the MATLAB (version R2024b, The MathWorks, Inc., Natick, MA, USA) Medical Imaging Toolbox, using the pre-trained weights of the ‘Medical Imaging Toolbox Model for Medical Segment Anything Model’ (version 24.2.0) without any further retraining or fine-tuning.

For a reliable application of the above segmentation methods, it is first necessary to perform effective image pre-processing to increase the visibility of the mass lesion. For the GVF active contour method, a specific pre-processing procedure has been defined, which is used to obtain the function *g* required to construct the edge map for all the mammograms under consideration. The procedure involves image normalization, followed by contrast adjustment, saturating values below 0.3 to 0 and values above 0.7 to 1. Finally, a local brightness enhancement is applied, increasing brightness in dark areas without saturating already bright regions. For Otsu thresholding, CVS and FCM methods, various combinations of pre-processing are tested. The images undergo a series of pre-processing steps, including normalization, contrast enhancement, noise reduction using Gaussian filters and morphological operations, as well as the application of principal component analysis (PCA). The combinations of the techniques used are listed and described in Table S2 of the Supplementary Materials, with Figure S2 showing the application of the different pre-processing steps to a sample image. Figure S2 also reports the corresponding edge map of the sample image for GVF active contour use. Conversely, MedSAM is applied to the image without any pre-processing, as done with Mask R-CNN. Specifically, the bounding box prompt provided to MedSAM corresponds exactly to the RoIs used as input for the other segmentation models.

### 2.5. Assessment via Segmentation Metrics

To assess the intersection between the predicted masks and the reference shapes, overlap-based metrics such as IoU, Dice similarity coefficient (DSC), Precision, Recall and Accuracy are considered [64]. These are defined according to the following relationships:

(5) $$\begin{array}{l} \mathrm{IoU}=\frac{\mathrm{TP}}{\mathrm{TP}+\mathrm{FP}+\mathrm{FN}},\;\mathrm{DSC}=\frac{2\mathrm{TP}}{2\mathrm{TP}+\mathrm{FP}+\mathrm{FN}},\;\mathrm{Precision}=\frac{\mathrm{TP}}{\mathrm{TP}+\mathrm{FP}},\\ \mathrm{Recall}=\frac{\mathrm{TP}}{\mathrm{TP}+\mathrm{FN}},\;\mathrm{Accuracy}=\frac{\mathrm{TP}+\mathrm{TN}}{\mathrm{TP}+\mathrm{TN}+\mathrm{FP}+\mathrm{FN}} \end{array}$$

where true positive (TP) corresponds to the pixels correctly predicted as part of the reference shape object, true negative (TN) to the background pixels correctly predicted as background, false positive (FP) to the background pixels incorrectly labeled as part of the reference shape, and false negative (FN) to the reference shape pixels that the model missed. These metrics scale from 0 (no overlap) to 1 (perfect overlap).

To quantify the discrepancy with respect to the ground-truth profiles, we evaluate distance-based metrics like the 95th percentile of the Hausdorff distance (HD95) and the average symmetric surface distance (ASSD) [69]. These are non-normalized metrics, and lower values indicate better segmentation performance. HD is the greatest of all the distances from a pixel *P* in the predicted mask boundary *B*_p_ to the closest pixel *Q* in the reference shape boundary *B*_r_ and vice versa, being defined as

(6) $$\mathrm{HD}=\max\left\{\underset{P\in B_{p}}{\sup}\underset{Q\in B_{r}}{\inf}d\left(P,Q\right),\underset{Q\in B_{r}}{\sup}\underset{P\in B_{p}}{\inf}d\left(P,Q\right)\right\}$$

ASSD is the average of all shortest Euclidean distances from pixels on the predicted mask surface *S*_p_ to the reference shape surface *S*_r_ and vice versa, namely:

(7) $$\mathrm{ASSD}=\frac{\sum_{P\in S_{p}}d\left(P,S_{r}\right)+\sum_{Q\in S_{r}}d\left(Q,S_{p}\right)}{N_{p}+N_{r}}$$

where *N*_p_ and *N*_r_ are the numbers of pixels belonging to *S*_p_ and *S*_r_, respectively.

As a distance-based metric, we also consider the normalized surface distance (NSD) to measure the spatial overlap between the predicted and reference boundaries. It provides an estimation of the fraction of boundary pixels in the predicted mask that fall within a specified tolerance distance (τ) from the reference shape boundary) [70]. This is defined as

(8) $$\mathrm{NSD}=\frac{\sum_{P\in S_{p}}\left[\min_{Q\in S_{r}}d\left(P,Q\right)\leq \tau \right]+\sum_{Q\in S_{r}}\left[\min_{P\in S_{p}}d\left(Q,P\right)\leq \tau \right]}{N_{p}\;+\;N_{r}}$$

Here, the tolerance distances are set at 0.5 mm (NSD05), 0.75 mm (NSD075), 1 mm (NSD1) and 2 mm (NSD2). NSD scales from 0 (no overlap) to 1 (perfect overlap).

## 3. Results

For a first evaluation of segmentation performance, the optimal configuration of Mask R-CNN incorporating a customized SGD optimizer and a StepLR scheduler (Model 3) is applied to a synthetic mammogram containing artificial lesions used as an objective ground truth. Then, it is investigated by performing an independent test on a set of real mammograms from CSAW-CC and TOMPEI-CMMDs. Finally, the model is compared to classical segmentation methods, the FCM clustering algorithm, and MedSAM, considering both quantitative metrics (based on overlap area and edge distance) and qualitative assessment by an expert breast cancer radiologist.

### 3.1. Segmentation of Artificial Lesions in a Synthetic Mammogram

**Figure 4:**
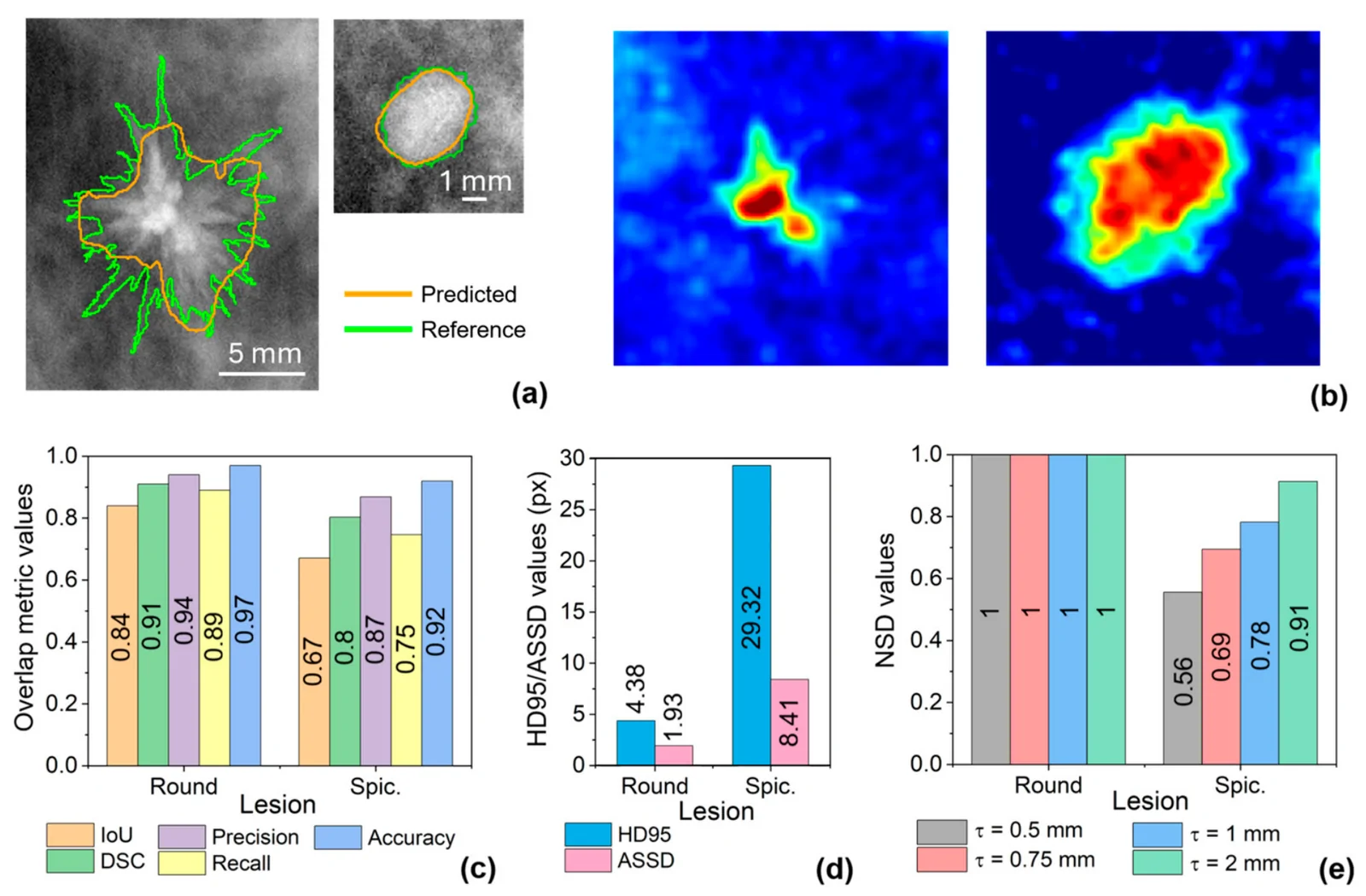
(**a**) Application of the Mask R-CNN model to a spiculated mass (**left**) and a round mass (**right**) in a synthetic mammogram generated with VICTRE [51]. The predicted contours are compared to the boundaries of the artificial lesion mask, used as ground truth. (**b**) Score-CAM heatmaps to visually explain the outputs of the customized lesion-detection CNN, used to assess synthetic lesion realism. The colour scale ranges from blue (low activation, non-lesion) to red (high activation, lesion). Evaluation of Mask R-CNN segmentation performance by means of (**c**) overlap-based metrics (IoU, DSC, Precision, Recall and Accuracy) and distance-based metrics (**d**) HD95 and ASSD and (**e**) NSD with tolerance distance τ equal to 0.5 mm, 0.75 mm, 1 mm and 2 mm.

The synthetic mammogram for the evaluation of segmentation accuracy of Mask R-CNN is generated ad hoc with the in silico breast imaging simulator VICTRE (Virtual Imaging Clinical Trials for Regulatory Evaluation) developed by the US Food and Drug Administration (FDA) [71,72]. We create a digital breast phantom with scattered regions of fibroglandular tissue, where we insert two types of mass lesions by means of a replacement of tissue at voxel level, overwriting the existing tissue label with a new distinct lesion label [73]. The lesions, generated with the breast mass generation software incorporated into VICTRE [74], correspond to a round mass with well-defined margins and to a spiculated mass with linear regions radiating outward (Figure 4a). The former has an average size of 4 mm, while the latter has an average size of 10 mm with spicules with length varying from 1 to 6 mm. After phantom biomechanical compression with the finite element biomechanics solver FEBio integrated in VICTRE [75], we simulate the interaction between X-rays and breast tissues and obtain a synthetic “for presentation” mammogram with a spatial resolution of 85 μm, reproducing the image processing of the Siemens Mammomat Inspiration system. The irradiation parameters and tissue mass densities used in the simulation can be found in [73].

The realism of the mass lesions is tested by means of a CNN customized as a binary lesion detection model, to classify mammogram patches into “Lesion” and “No Lesion” categories [76]. To ensure a consistent input size as required by the CNN architecture, all patches are resized to 300 × 300 px using Lanczos interpolation, which guarantees the preservation of fine details and sharp edges during resizing. The CNN was trained and validated on real mammograms, including Siemens images from VinDr-Mammo and OMI-DB (1806 for training and 392 for validation) and Hologic images from CSAW and OMI-DB (12,990 for training and 2784 for validation); half of the extracted patches contain annotated lesions. Tested on 3170 real mammograms balanced between healthy and diseased cases, the network achieved a classification confidence of 94%, with 99% true positive and true negative rates. When applied to the two image patches extracted from the synthetic mammogram, the CNN classifies them into the “Lesion” category with confidence scores of 83.1% for the spiculated mass and 99.1% for the well-defined mass. To highlight the regions recognized as “Lesion”, Figure 4b displays the corresponding heatmaps calculated via the score-weighted class activation mapping (Score-CAM) visual explanation method [77].

Having demonstrated the realism of the artificial lesions, we generate the corresponding segmentation masks using the *get_coordinates_dm(vx_location)* function implemented in VICTRE. This function maps a 3D voxel coordinate from the simulated breast phantom space (within the mass lesion volume) to its corresponding 2D pixel coordinate on the projection image plane [72,78]. To generate the mask of the spiculated mass, we include only lesion portions containing more than 5 voxels along the projection direction. This strategy is chosen to avoid deriving too sharp mask edges and to exclude image areas where the lesion is not clearly visible, as very few lesion voxels traversed by the X-ray produce minimal impact on pixel intensity and can easily be masked by image noise. The two obtained masks, highlighted by the green lines in Figure 4a, can be used as ground truth for the evaluation of the accuracy of the Mask R-CNN model in the detection of breast mass margins, using the quantitative metrics listed in Section 2.5. The segmentation results are overlaid on Figure 4a using orange lines.

The Mask R-CNN model can accurately detect the contours of the round mass, with metric scores closer to the target value than those obtained for the spiculated lesion (Figure 4c–e). Specifically, the round mass shows a high mask agreement with the ground truth, with IoU and DSC scores of 0.84 and 0.91, respectively, compared with significantly lower values for the spiculated lesion (IoU = 0.67, DSC = 0.80). Nevertheless, for the spiculated lesion, Accuracy, Precision and Recall remain at satisfactory levels, equal to 0.92, 0.87 and 0.75, respectively; these values do not necessarily indicate perfect delineation of the contour, but they highlight the model’s ability to correctly identify the position and overall extent of the lesion within the surrounding tissue. The observed morphological discrepancy is supported by an analysis of the distance-based metrics: for the round lesion, the HD95 and ASSD values amount to 4.38 px (equivalent to 0.37 mm) and 1.93 px (0.16 mm), respectively, indicating a marginal localization error compared with the much higher values of 29.32 px (2.48 mm) for HD95 and 8.41 px (0.71 mm) for ASSD, estimated for the spiculated mass. Finally, the NSD values confirm optimal geometric accuracy for the round mass, achieving the maximum score (1) even at the lower tolerance of 0.5 mm. For the spiculated lesion, the irregularity of the margins is responsible for a drastic reduction in the NSD to 0.56 for τ = 0.5 mm, requiring higher tolerances to obtain acceptable profile overlap; NSD is equal to 0.70 for τ = 0.75 mm, and reaches a value of 0.91 for τ = 2 mm.

### 3.2. Segmentation of Real Lesions in Clinical Mammograms

**Figure 5:**
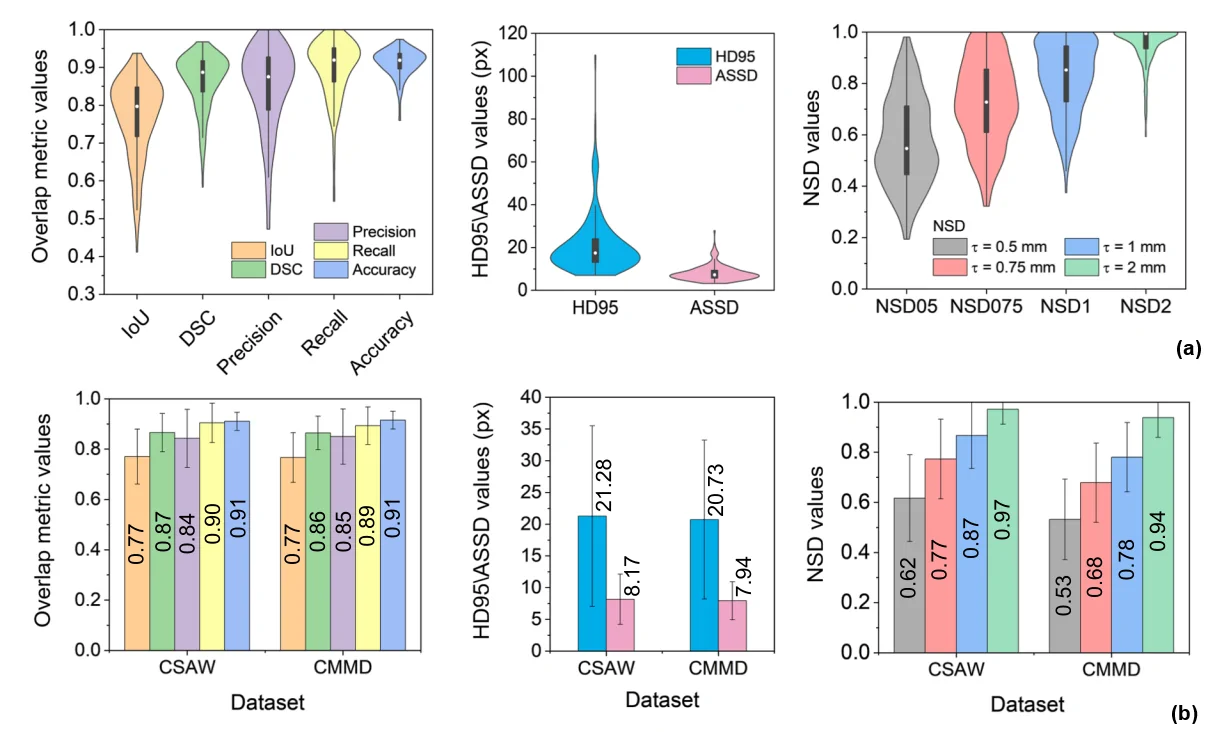
Evaluation of segmentation performance of Mask R-CNN when applied to a set of 215 real mammograms; the predicted masks are compared to the annotated masks provided by CSAW-CC and TOMPEI-CMMD, used as reference. (**a**) Violin plots illustrating the metric scores for the full test set; (**b**) bar charts displaying the metric means and standard deviations, distinguished by database source. Left: overlap-based metrics (IoU, DSC, Precision, Recall and Accuracy); center: distance-based metrics HD95 and ASSD; right: distance-based metric NSD with τ equal to 0.5 mm, 0.75 mm, 1 mm and 2 mm.

Mask R-CNN is tested here on an independent set of 215 RoIs extracted from clinical mammograms where both round and spiculated mass lesions are present; this comprises 112 patches selected from CSAW-CC and 103 from TOMPEI-CMMD, not contained in the training and validation sets. The model has proved to detect the lesions in all the extracted patches, and its segmentation performance is measured considering both overlap- and distance-based metrics, calculated by comparing the model outputs to the annotated masks provided by the two databases. Figure 5a shows the metric distributions via violin plots, combining the results obtained for the two datasets, while the bar charts in Figure 5b report metric means and standard deviations separated for each database.

Overall, most of the overlap-based metrics exceed 0.70. Notably, Accuracy and Recall achieve the highest average scores, 0.91 and 0.90, respectively, highlighting the network’s effectiveness in correctly segmenting the lesion within the patch. Conversely, IoU and Precision record the lowest average scores, 0.77 and 0.85, respectively, and exhibit greater variability, with lower outliers ranging from 0.40 to 0.50. The DSC shows an intermediate behavior with an average of 0.87, but its lower tail is shifted toward higher values compared to IoU, Precision, and Recall, with minima that always remain above 0.58.

Looking at individual scores across the entire test set, 51 predicted masks achieve high intersection performance, with all the overlap-based metrics exceeding 0.85 (31 from CSAW-CC and 20 from TOMPEI-CMMD). Notably, this includes 10 cases that consistently score above 0.9 (4 from CSAW-CC and 6 from TOMPEI-CMMD). Additionally, there are 18 low-overlap cases where both IoU and Precision fall below 0.6, though only 3 drop below 0.5. These instances share distinct characteristics: the lesions are small, with an average diameter of approximately 3 mm, and exhibit poor contrast against surrounding tissues. This leads to an overestimation of the lesion areas, with a significant number of false-positive pixels that degrade the evaluation metrics. The bar chart of the overlap-based metrics shows a similar trend for the two datasets, with nearly equivalent means and standard deviations for all metrics. This similarity is confirmed by a Wilcoxon rank-sum test, which reveals no significant differences between the overlap-based metric distributions of the two databases, as detailed in Table S3.

Considering the distance-based metrics, NSD values rise sharply as the tolerance distance τ increases, from 0.58 at τ = 0.5 mm to 0.96 at τ = 2 mm, demonstrating that most contour errors lie within 0.5 mm of the reference boundary. Across all tolerances, TOMPEI-CMMD consistently yields lower NSD values than CSAW-CC: 0.53 vs. 0.62 (τ = 0.5 mm), 0.68 vs. 0.77 (τ = 0.75 mm), 0.78 vs. 0.87 (τ = 1 mm), and 0.94 vs. 0.97 (τ = 2 mm). The discrepancy between the segmentation outputs of the images from the two databases is particularly evident when comparing HD95 and ASSD values in millimeters, where the highest errors are found for the mammograms from TOMPEI-CMMD. Specifically, TOMPEI-CMMD yields an HD95 mean of 1.95 mm (20.7 px) compared to 1.49 mm (21.3 px) for CSAW-CC. Similarly, the ASSD mean is 0.75 mm (7.9 px) for TOMPEI-CMMD and 0.57 mm (8.2 px) for CSAW-CC. The distributions of HD95 and NSD (for τ ≤ 1 mm) are the most dispersed. While ASSD is averaged over the entire contour and remains less sensitive to localized discrepancies, HD95 captures the worst-case error. Consequently, HD95 exhibits pronounced outliers, including a maximum value of 110 px (approximately 7.7 mm). This extreme value is attributable to a remarkably large lesion with an equivalent diameter of roughly 63 mm, as illustrated in Figure S3 of the Supplementary Materials. Anyway, the Wilcoxon rank-sum test proves that the differences between TOMPEI-CMMD and CSAW-CC are significant only for the NSD metrics, resulting in negligible differences for HD95 and ASSD (Table S3).

**Figure 6:**
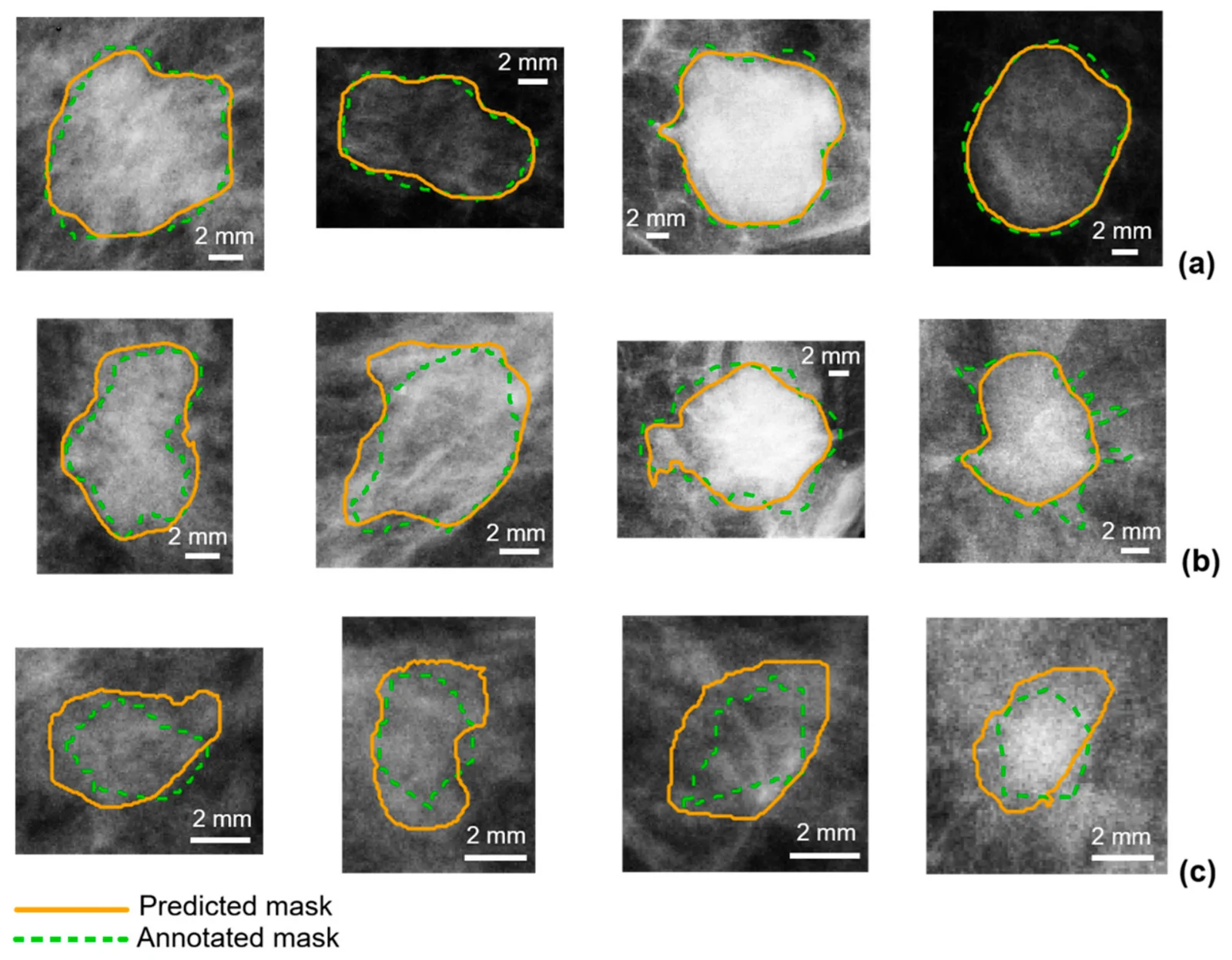
Representative examples of Mask R-CNN segmentation results (orange curves) in comparison to ground-truth annotations (in green dashed curves) provided by CSAW-CC and TOMPEI-CMMD, with scale bars indicating spatial dimensions in millimeters. (**a**) High-performance cases (DSC > 0.85), (**b**) medium-performance cases exhibiting Precision/Recall trade-offs, and (**c**) low-performance cases characterized by small and low-contrast lesions. Each row comprises four image patches sourced from CSAW-CC [(**a**,**b**): first two columns; (**c**): first three columns] and TOMPEI-CMMD [(**a**,**b**): last two columns; (**c**): fourth column]. Further details about source mammograms can be found in Table S8 of the Supplementary Materials.

Representative examples demonstrating various segmentation behaviors and performance trade-offs are illustrated in Figure 6, with the corresponding metric scores reported in Table S4. Figure 6a depicts four representative cases selected from the high-performance segmentations (i.e., overlap-based metrics consistently exceeding 0.85–0.90), demonstrating high accuracy in boundary delineation and lesion localization. Figure 6b displays four examples with more varied metric values, showing an asymmetric trade-off between Precision and Recall. In the two cases on the left, the lesion area is overestimated, yielding higher Recall than Precision (e.g., Precision = 0.79 vs. Recall = 0.98 for the first lesion). Conversely, the two cases on the right exhibit a general underestimation (higher Precision than Recall), particularly in the presence of spicules (e.g., Precision = 0.97 vs. Recall = 0.82 for the third lesion). Despite these trade-offs, performance remains robust; all metrics exceed 0.75 (reaching up to 0.98 for Recall), and Accuracy remains greater than or equal to 0.90 across all cases.

Marked over-segmentation is reported in Figure 6c, which features lower-performance cases characterized by a shared condition: small lesions with low contrast relative to the surrounding tissues. A clear Precision/Recall trade-off is also observed here; for instance, the first lesion on the left yields a Precision of 0.56 and a Recall of 0.91. However, even in these challenging cases, Accuracy remains high, ranging between 0.84 and 0.90.

Comparing Figure 6a,b, the variation in overlap-based metrics is accompanied by a significant increase in distance-based metrics. Specifically, the mean of HD95 rises from 9.4 px in Figure 6a to 15.8 px in Figure 6b, while the mean of ASSD increases from 4.4 px to 7 px. Interestingly, in the worst-performance cases (Figure 6c), HD95 (averaging 13.7 px) and ASSD (averaging 7.1 px) do not exhibit further substantial degradation despite a sharp drop in IoU and DSC. This behavior is directly attributable to the small size of the lesions in Figure 6c, which inherently restricts the absolute spatial displacement of the predicted contour. This effect is further substantiated by the NSD values; while a strict tolerance threshold (τ = 0.5 mm) highlights contour discrepancies in small lesions (dropping as low as 0.35), broader thresholds (NSD1 and NSD2) approach 1. This confirms that the over-segmentation errors occur within a very narrow pixel radius with respect to the ground-truth contours.

To evaluate the impact of lesion size on segmentation performance, metric distributions from both databases are combined and stratified into tertiles based on the annotated lesion equivalent diameter. Lesions are categorized into three distinct groups: small (<10 mm), medium (10–15 mm), and large (>15 mm), with the corresponding box plots presented in Figure S4. Because most of the performance metrics exhibit a non-normal distribution, the Spearman rank correlation coefficient (ρ) is calculated to assess the association between each metric and lesion diameter. A Kruskal–Wallis test is performed to evaluate statistically significant differences in performance across the three size categories (Table S5). The analysis shows a positive and significant Spearman correlation between lesion diameter and the metrics IoU, DSC, and Precision (with ρ equal to 0.41, 0.41, and 0.45, respectively), confirming the small object penalty effect, i.e., segmentation performance decreases as lesion diameter diminishes. This finding is corroborated by the Kruskal–Wallis test, which indicates significant differences across size tertiles. Conversely, no significant correlation is found for Recall and Accuracy, with no meaningful differences across tertiles, suggesting that the model’s segmentation capability remains stable regardless of lesion size. Finally, a moderate positive correlation is observed for HD95 and ASSD (ρ equal to 0.47 and 0.49, respectively), consistent with a negative correlation for all NSD metrics (ρ ranging from −0.51 to −0.45), meaning that normalized surface distance worsens as size increases. The Kruskal–Wallis test confirms significance for both groups of distance-based metrics. Overall, the obtained results indicate that while overlap-based metrics (IoU, DSC, Precision) benefit from larger lesion sizes, the distance-based metrics can degrade for larger regions.

### 3.3. Comparison with Other Segmentation Methods on Real Mammograms

The performance of the optimal configuration of Mask R-CNN (Model 3) is finally evaluated by comparison to the segmentation methods described in Section 2.4, considering a subset of 155 images (55 from CSAW-CC and 100 from TOMPEI-CMMD) extracted from the test dataset used in Section 3.3. The masks provided by CSAW-CC and TOMPEI-CMMD are again leveraged as a reference. The Otsu thresholding, CVS and FCM methods are applied to the mammograms pre-processed with all the pre-treatment approaches listed in Table S2 of the Supplementary Materials, but for the image-level comparison, we only consider the specific method that yields the best segmentation outputs for each individual image. Further details are provided in Figure S5, where the histograms report the number of image patches for which each pre-processing technique yields superior performance based on the average of overlap-based metrics. It can be observed that there is no single optimal pre-processing that works well for all images, because of the variations in contrast, sharpness and lesion features across images. Pre-processing pipeline ID 25 (intensity-level quantization followed by morphological opening on the normalized image) most frequently yields the best results for the Otsu thresholding, CVS and FCM methods.

**Figure 7:**
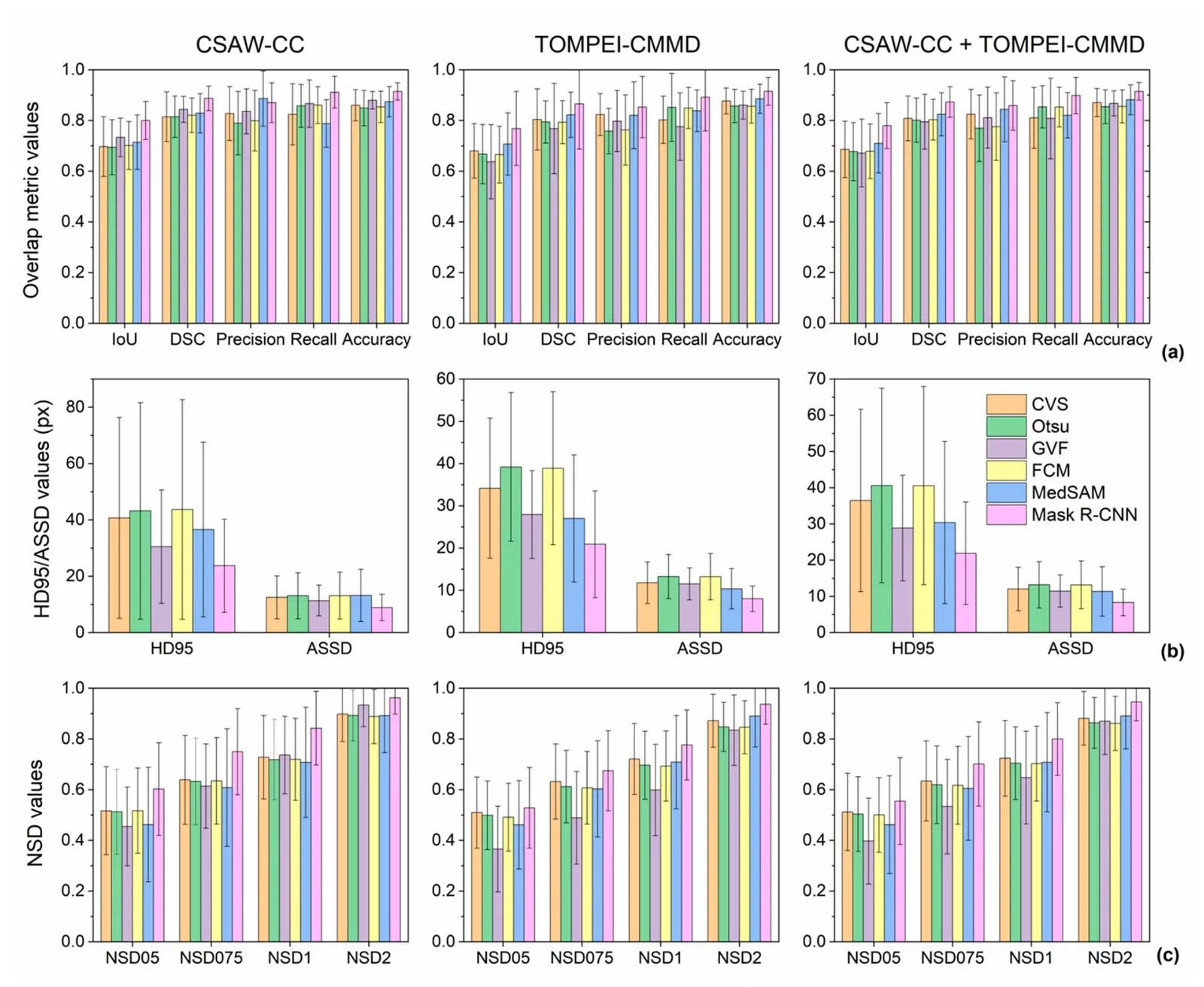
Application of Mask R-CNN model and other segmentation methods (CVS, Otsu thresholding, GVF active contour, FCM and MedSAM) to a set of 155 real mammograms; the predicted masks are compared to the annotated masks provided by CSAW-CC and TOMPEI-CMMD, used as reference. Evaluation of segmentation performance by means of (**a**) overlap-based metrics (IoU, DSC, Precision, Recall and Accuracy), distance-based metrics (**b**) HD95 and ASSD, and (**c**) NSD with τ equal to 0.5 mm, 0.75 mm, 1 mm and 2 mm. From left to right, the graphs report the results for the images from CSAW-CC, TOMPEI-CMMD, and the two combined datasets, respectively.

The means and standard deviations of all the metrics are shown in the bar charts of Figure 7, with the overlap-based metrics in Figure 7a, the HD95 and ASSD scores in Figure 7b, and the NSD values for tolerance distance τ of 0.5, 0.75, 1 and 2 mm in Figure 7c. The distributions of the metrics are illustrated by the violin plots in Figure S6 of the Supplementary Materials. The outputs confirm a coherent trend for all the segmentation methods; among the overlap-based metrics, IoU always reaches the lowest values, while Accuracy reaches the highest; NSD increases markedly as tolerance increases, ranging from about 0.5 for τ = 0.5 mm to over 0.9 for τ = 2 mm. ASSD exhibits low values, while HD95 scores are significantly high and associated with a large standard deviation; this means that the mask boundary predictions are very similar to the reference ones on average, but suffer from a few severe outliers, as demonstrated by the violin plots in Figure S6.

Overall, the Mask R-CNN model outperforms the other methods, particularly Otsu thresholding, CVS, and FCM, with the highest values for the overlap-based and NSD metrics, and the lowest and most stable scores for HD95 and ASSD. The HD95 values indicate greater variability in contour definition for FCM and the classical methods, with particularly high values for CVS and Otsu thresholding. This suggests that the Mask R-CNN model not only accurately estimates the overall size of the lesion but also generates contours that more faithfully capture its spatial distribution. Although GVF active contour stands out among classical methods as the most robust alternative for HD95 and ASSD, it is limited by substantial variability and low scores regarding NSD for τ lower than 1 mm. Furthermore, GVF active contour is highly sensitive to the initial contour settings, which must be configured manually; in contrast, for CVS, it is sufficient to select a small square of just a few pixels at the center of the RoI. Otsu thresholding shows the lowest Precision values, confirming a marked tendency toward over-segmentation. FCM exhibits results that are essentially comparable to those of Otsu thresholding, while MedSAM is the model that performs closest to Mask R-CNN. Specifically, MedSAM achieves a higher Precision score on the CSAW-CC dataset; in all other cases, Mask R-CNN obtains better scores, particularly for the distance-based metrics HD95 and ASSD, demonstrating superior performance in delineating lesion contours.

**Figure 8:**
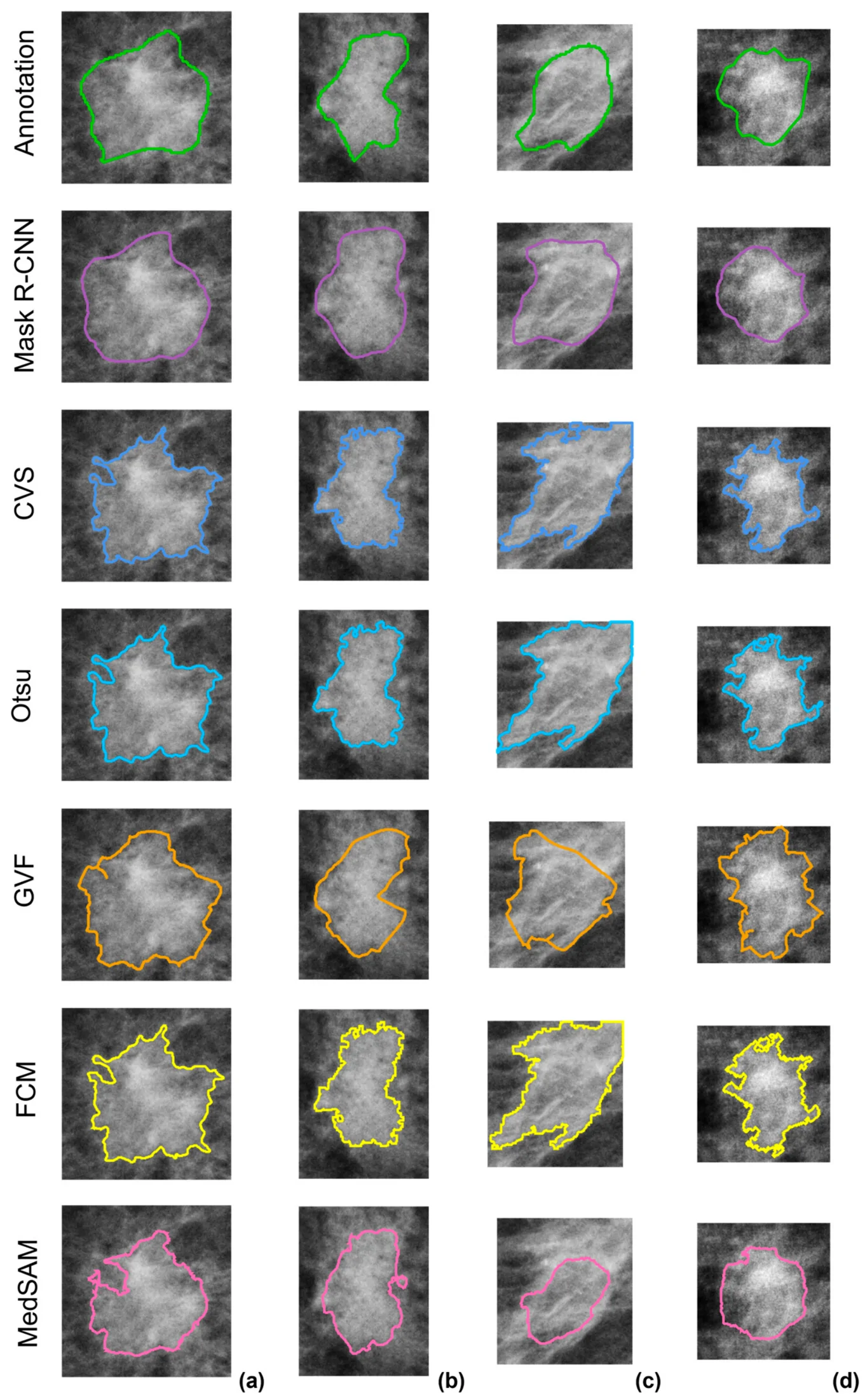
Application of Mask R-CNN model and other segmentation methods (CVS, Otsu thresholding, GVF active contour, FCM and MedSAM) to a set of real mammograms; the segmentation results are compared to the annotations provided by CSAW-CC and TOMPEI-CMMD, used as reference. Each column refers to a specific image patch; (**a**–**c**) are sourced from CSAW-CC, and (**d**) from TOMPEI-CMMD. Further details can be found in Table S8 of the Supplementary Materials.

Some segmentation examples are shown in Figure 8, where the contours obtained with the Mask R-CNN model and the other methods are compared with the reference masks (the corresponding metrics are listed in Table S6 of the Supplementary Materials). Mask R-CNN tends to produce smoother and more regular contours, while Otsu thresholding, CVS and FCM follow local variations in grayscale with greater pixel-by-pixel accuracy, capturing fine details but sometimes at the expense of the contour global consistency. For the mass lesions shown in Figure 8a,b, CVS and Otsu thresholding lead to distinct boundaries that closely align with the lesion actual structure than the smoother contours generated by Mask R-CNN. The metrics listed in Table S6 quantitatively confirm this observation for the segmentation results in Figure 8b, while they indicate that Mask R-CNN is the more accurate method for the lesion in Figure 8a. Notably, this lesion is well segmented by all methods except for MedSAM, which exhibits greater sensitivity to gradient changes in the upper-left region of the lesion.

For some methods, the tendency to focus on local details can lead to systematic errors of opposite signs. Specifically, it causes over-segmentation in Otsu thresholding, CVS and FCM, as shown in Figure 8c, where the contours extend well beyond the actual margins of the lesion, including surrounding tissue structures and reaching the patch edges. Otsu thresholding presents the lowest IoU score (0.34), followed by CVS and FCM with values of 0.57 and 0.59, respectively. Slightly higher values are observed for MedSAM (0.62) and GVF (0.69), while the best result is achieved by Mask R-CNN, with IoU equal to 0.76. Under-segmentation can also occur, as seen in Figure 8d, where the contours remain narrower than the reference mask boundary, excluding peripheral portions of the lesion. Furthermore, the GVF active contour exhibits distinct limitations in heterogeneous and low-contrast regions; this behavior is evident in Figure 8c, where the generated boundary deviates substantially from the actual morphology of the lesion. Mask R-CNN also struggles to segment this lesion, overestimating the annotated mask across several regions; MedSAM, by contrast, significantly underestimates it.

Additional segmentation examples qualitatively assessed by the co-author from the A.O.U. Città della Salute e della Scienza di Torino (Italy), an expert breast cancer radiologist, are reported in Figures S7 and S8 of the Supplementary Materials. According to the radiologist’s analysis, Mask R-CNN provides segmentation results that accurately capture the overall dimensions and boundaries of the lesions, without producing excessively jagged contours. This outcome is consistent with clinical annotation practices, where precise boundary delineation of minor local irregularities at pixel level is secondary to identifying the whole lesion shape and size, relevant for diagnostic purposes. Compared to other segmentation methods, Mask R-CNN demonstrates superior performance even in the case of low-contrast mass lesions, which typically occur when the surrounding tissue is predominantly fibroglandular, resulting in a similar radio-opacity. This is clear in Figure S7, where MedSAM exhibits greater difficulty segmenting lesions than Mask R-CNN.

## 4. Discussion

The segmentation results achieved in this study, quantified by means of overlap- and distance-based metrics, demonstrate that the optimal configuration of Mask R-CNN (Model 3) effectively captures the shape, size and spatial distribution of breast mass lesions, exhibiting consistent performance across both the used image source databases (CSAW-CC and TOMPEI-CMMD). Notably, Accuracy remains close to the target value even for classical segmentation methods, whereas other metrics yield less optimal scores. This discrepancy highlights that Accuracy offers limited discriminatory power; although this continues to be reported in some recent breast cancer segmentation studies [1], DSC and IoU provide significantly more informative assessments and remain the preferred standards in the contemporary literature.

Focusing on DSC and IoU, the performance achieved by Mask R-CNN in this study aligns with the current state of the art. For instance, on mammograms from the CBIS-DDSM database [23,26], architectures based on standard or enhanced U-Net models exhibit moderate performance, with DSC ranging from 0.72 to 0.81 and IoU from 0.57 to 0.65 [27,28]. More competitive results are reported for the Connected-UNets models by Baccouche et al. (maximum DSC and IoU of 0.85 and 0.74, respectively) [29] and for the end-to-end framework proposed by Li et al. (DSC of 0.92) [30]. A superior performance trend emerges when the models in [29,30] are tested on mammograms from the INbreast database [17], for which DSC values in the order of 0.95 are achieved. Similar results were obtained with INbreast using a multi-level nested pyramid network (MNPNet) [31], a multi-scale FCN [32], and an update of U-Net integrating residual learning and probabilistic graphical modeling [33], with DSC equal to or higher than 0.9. However, these reference studies rely on semantic segmentation models, whereas only a limited number of works have applied instance segmentation to mammography. Instance segmentation models tend to exhibit lower performance metrics in comparison to their semantic segmentation counterparts, since the additional task of object detection permits the examination of larger RoIs, even the whole mammograms, but at the expense of reduced segmentation metrics. This is well exemplified by the model proposed by Zambrano et al. [34], which achieved a DSC of 0.72 on INbreast. Among other studies applying instance segmentation, Min et al. employed Mask R-CNN to develop a CAD system for pseudo-colored mammograms (from INbreast), achieving a DSC of 0.88 and a true positive rate (TPR) of 0.90 [35]. Similarly, a Mask R-CNN-based model applied in breast MRI yields a DSC of 0.82, computed on true positives standardized via IoU [42].

To provide a clearer comparison between our study and these prior works, we also test the optimal configuration of Mask R-CNN (Model 3), trained on CSAW-CC and TOMPEI-CMMD, on all the INBreast images for which precise lesion annotations are available (113), finding an average DSC of 0.89. This result aligns well with the existing instance segmentation literature, matching the performance reported by Min et al. [35] and outperforming the model proposed by Zambrano et al. [34]. Notably, the considered INbreast images were acquired with Siemens scanners and thus refer to a vendor style unseen in the training phase, as the CSAW-CC and TOMPEI-CMMD datasets consist of images from Hologic and GE HealthCare, respectively. This demonstrates the model’s generalization to unobserved imaging systems and its capacity to adapt to different vendor styles, which can differ substantially in overall image appearance [15]. Examples of segmentation contours generated with Mask R-CNN on INbreast images are shown in Figure S9 of the Supplementary Materials, with the scores obtained for all metrics in Table S7.

While the comparison above of models from the literature relies strictly on traditional overlap-based metrics, a key contribution of our study is the inclusion of distance-based metrics. Although still underutilized, these metrics prove essential to our benchmarking, as they clearly demonstrate the superiority of Mask R-CNN over classical segmentation methods, clustering algorithms like FCM and the SOTA model MedSAM, a distinction that overlap-based metrics alone tend to obscure. Furthermore, edge-based evaluation reveals that the quality of contour reconstruction is highly dependent on lesion morphology. Mask R-CNN achieves optimal pixel-level localization for masses with round or oval margins. Conversely, for lesions with spiculated and irregular contours, the reproduction of fine details is less accurate, even though the primary mass is consistently and correctly localized. Notably, the advantage of Mask R-CNN is most pronounced in low-contrast images, where dense fibroglandular tissue renders the lesion difficult to distinguish from the background. In these challenging scenarios, classical methods and FCM frequently fail, whereas Mask R-CNN maintains robust segmentation performance comparable to that observed in more favorable cases, where the background consists primarily of adipose tissue, and the lesion is clearly visible.

Another aspect worth noting is that the optimal configuration of Mask R-CNN tested in our work can achieve high accuracy despite a relatively small volume of training and validation images. This is made possible through transfer learning and the use of accurately annotated databases with high-precision lesion segmentation. Beyond these architectural strengths, our study further contributes a novel strategy for model validation through the leveraging of artificial lesions within synthetic mammograms. Because their spatial distribution and features are precisely known, this approach effectively eliminates the biases typically associated with inter-observer variability in radiological annotations.

Regarding model limitations, Mask R-CNN accuracy drops when reproducing the fine details of spiculated lesions. Addressing this issue requires enriching the training datasets with more examples of these complex morphologies; however, spiculated masses suffer from limited availability in standard, publicly accessible screening mammography databases. Moreover, the model training phase remains constrained by manual radiological annotations, which are intrinsically subject to human errors and inter-observer variability. This constraint could be effectively bypassed by incorporating ground-truth synthetic data directly into the training pipeline, rather than utilizing it solely as a downstream validation tool. Finally, although the instance segmentation approach offers the distinct advantage of simultaneous object detection and localization, this added task inherently trades off absolute pixel-level precision, resulting in metrics that remain slightly lower than those typically reported for highly optimized semantic segmentation frameworks.

## 5. Conclusions

This study investigates the Mask R-CNN deep learning model for the segmentation of mass lesions in mammography images. The optimal model configuration, derived through the training and validation pipeline, incorporates a customized SGD optimizer and a StepLR scheduler. The application of this configuration to patches extracted from real mammograms belonging to the CSAW-CC and TOMPEI-CMMDs yields high accuracy. The model correctly delineates lesion contours, achieving an average DSC of 0.87 and an average IoU of 0.77, outperforming conventional segmentation methods, clustering algorithms like FCM and the SOTA model MedSAM. The obtained results align with the current state of the art for CNN-based medical image segmentation, demonstrating robust generalization even when the model is applied to images with unseen vendor styles, such as those in the INbreast database. Coupled with very low inference times, these findings confirm that Mask R-CNN is a highly viable tool for integration into computer-aided diagnosis workflows.

## Data Availability

The data presented in this study are available from the corresponding authors on reasonable request. The study uses, as data sources, publicly archived datasets, namely CSAW-CC (https://www.kaggle.com/datasets/ahmadihossein/csaw-cc-mammography, accessed on 1 October 2026), TOMPEI-CMMD (https://www.cancerimagingarchive.net/analysis-result/tompei-cmmd/, accessed on 1 October 2026) and INbreast (https://www.kaggle.com/datasets/ramanathansp20/inbreast-dataset, accessed on 1 October 2026).

## Supplementary Materials

The following supporting information can be downloaded at: https://www.mdpi.com/article/10.3390/diagnostics16203289/s1, Table S1: Mask R-CNN hyperparameters and model information corresponding to configuration Model 3; Figure S1: comparison of Mask R-CNN model across three different seeds, in terms of training/validation loss, BBox AP, and Segm AP; Table S2: list of applied pre-processing pipelines for Chan–Vese segmentation, Otsu thresholding and Fuzy c-means algorithm; Figure S2: application of image pre-processing pipelines on a sample image; Table S3: Wilcoxon rank-sum test results comparing segmentation metrics across database sources; Figure S3: comparison between Mask R-CNN segmentation and the reference mask for a lesion with the highest Hausdorff distance value; Table S4: metric values calculated for the images in Figure 6; Figure S4: box plots of metric distributions evaluated using Mask R-CNN for the TOMPEI-CMMD and CSAW databases, categorized into tertiles by lesion diameter; Table S5: correlation analysis of the metrics evaluated with Mask R-CNN with respect to lesion size; Figure S5: histograms showing the number of image patches for which each pre-processing technique leads to superior performance in Chan–Vese segmentation, Otsu thresholding and Fuzy c-means algorithm; Figure S6: violin plots of metric distribution comparing R-CNN model with selected segmentation methods; Table S6: metric values calculated for the images in Figure 8; Figures S7 and S8: application of Mask R-CNN and other segmentation methods to real mammograms from the TOMPEI-CMMD and CSAW-CC databases, respectively; Figure S9: application of Mask R-CNN to real mammograms from the INbreast database; Table S7: metrics values calculated with Mask R-CNN model to INbreast database; Table S8: cetails regarding each mammogram from which the patches for the reported experimental analysis are extracted.

## Abbreviations

The following abbreviations are used in this manuscript:

AP	Average Precision
ASSD	Average Symmetric Surface Distance
BBox AP	Bounding Box Average Precision
CAD	Computer-Aided Diagnosis
CBIS-DDSM	Curated Breast Imaging Subset of Digital Database for Screening Mammography
CMMD	Chinese Mammography Database
CNN	Convolutional Neural Networks
COCO	Common Objects in Context
CSAW-CC	Swedish Cohort of Screen-Age Women Case–Control
CPU	Central Processing Unit
CVS	Chan–Vese Segmentation
DCE-MRI	Dynamic Contrast-Enhanced Magnetic Resonance Imaging
DICOM	Digital Imaging and COmmunications in Medicine
DSC	Dice Similarity Coefficient
EMBED	US EMory BrEast imaging Dataset
Faster R-CNN	Faster Region-based Convolutional Neural Network
FCN	Fully Convolutional Network
FC	Fully Connected
FN	False Negative
FP	False Positive
FPN	Feature Pyramid Network
GPU	Graphics Processing Unit
GVF	Gradient Vector Flow
HD	Hausdorff Distance
HD95	95th Percentile of the Hausdorff Distance
IoU	Intersection over Union
LR	Learning Rate
Mask R-CNN	Mask Region-based Convolutional Neural Network
MRI	Magnetic Resonance Imaging
NSD	Normalized Surface Dice
OMI-DB	OPTIMAM Medical Image Database
PCA	Principal Component Analysis
RAM	Random Access Memory
RPN	Region Proposal Network
ResNet	Residual Network
RoI	Region of Interest
Segm AP	Segmentation Average Precision
SGD	Stochastic Gradient Descent
TN	True Negative
TP	True Positive
U-Net	U-shaped Network
VICTRE	Virtual Imaging Clinical Trials for Regulatory Evaluation
VinDr-Mammo	Vietnamese Dataset of Digital Mammography

## References

[1] Fajrin H.R., Min S.D. (2026). An Overview of Segmentation Techniques in Breast Cancer Detection: From Classical to Hybrid Model. Comput. Mater. Contin..

[2] Michael E., Ma H., Li H., Kulwa F., Li J. (2021). Breast Cancer Segmentation Methods: Current Status and Future Potentials. BioMed Res. Int..

[3] Amin A., U D.A., Koteshwara P., P C S., Mathew S. (2025). A Systematic Literature Review on Mammography: Deep Learning Techniques for Breast Cancer Detection with Global and Asian Perspectives. BMC Cancer.

[4] Avcı H., Karakaya J. (2024). Segmentation Techniques in Mammography: Their Value in Breast Cancer Diagnosis. J. Clin. Pract. Res..

[5] Cheng H.D., Shi X.J., Min R., Hu L.M., Cai X.P., Du H.N. (2006). Approaches for Automated Detection and Classification of Masses in Mammograms. Pattern Recognit..

[6] Jyothisree K.R., Jacob V. (2025). Automated Breast Cancer Diagnosis via Intensity-Guided Contour Segmentation and ResNet-Based Classification Using Local Intensity Pattern Tensors. Sci. Rep..

[7] Ahmad S., Neal Joshua E.S., Rao N.T., Ghoniem R.M., Taye B.M., Bharany S. (2025). A Multi Stage Deep Learning Model for Accurate Segmentation and Classification of Breast Lesions in Mammography. Sci. Rep..

[8] Ravikumar M., Rachana P.G., Shivaprasad B.J. (2023). Segmentation of Tumour from Mammogram Images Using U-SegNet: A Hybrid Approach. Comput. Methods Biomech. Biomed. Eng. Imaging Vis..

[9] Li S., Dong M., Du G., Mu X. (2019). Attention Dense-U-Net for Automatic Breast Mass Segmentation in Digital Mammogram. IEEE Access.

[10] Zeiser F.A., Da Costa C.A., Zonta T., Marques N.M.C., Roehe A.V., Moreno M., Da Rosa Righi R. (2020). Segmentation of Masses on Mammograms Using Data Augmentation and Deep Learning. J. Digit. Imaging.

[11] Kalpana V., Thilagamani S. (2026). TOMPEI-CMMD: An adaptive radiological feature analysis using unitary U-net for high-precision mammography segmentation. Neurocomputing.

[12] Wang Y., Ali M., Mahmood T., Rehman A., Saba T. (2025). Robust Bi-CBMSegNet Framework for Advancing Breast Mass Segmentation in Mammography with a Dual Module Encoder-Decoder Approach. Sci. Rep..

[13] Saini V., Khurana M., Challa R.K. (2025). A Hybrid Model for the Segmentation of Mammogram Images Using Otsu Thresholding, Morphology and U-Net. Biomed. Pharmacol. J..

[14] Nour A., Boufama B. (2025). Hybrid Deep Learning and Active Contour Approach for Enhanced Breast Lesion Segmentation and Classification in Mammograms. Intell.-Based Med..

[15] Sharma N., Ng A.Y., James J.J., Khara G., Ambrózay É., Austin C.C., Forrai G., Fox G., Glocker B., Heindl A. (2023). Multi-Vendor Evaluation of Artificial Intelligence as an Independent Reader for Double Reading in Breast Cancer Screening on 275,900 Mammograms. BMC Cancer.

[16] Heath M., Bowyer K., Kopans D., Moore R., Kegelmeyer P. The Digital Database for Screening Mammography. Proceedings of the Fifth International Workshop on Digital Mammography.

[17] Moreira I.C., Amaral I., Domingues I., Cardoso A., Cardoso M.J., Cardoso J.S. (2012). INbreast. Acad. Radiol..

[18] Dembrower K., Lindholm P., Strand F. (2020). A Multi-Million Mammography Image Dataset and Population-Based Screening Cohort for the Training and Evaluation of Deep Neural Networks—The Cohort of Screen-Aged Women (CSAW). J. Digit. Imaging.

[19] Nguyen H.T., Nguyen H.Q., Pham H.H., Lam K., Le L.T., Dao M., Vu V. (2023). VinDr-Mammo: A Large-Scale Benchmark Dataset for Computer-Aided Diagnosis in Full-Field Digital Mammography. Sci. Data.

[20] Cai H., Wang J., Dan T., Li J., Fan Z., Yi W., Cui C., Jiang X., Li L. (2023). An Online Mammography Database with Biopsy Confirmed Types. Sci. Data.

[21] Jeong J.J., Vey B.L., Bhimireddy A., Kim T., Santos T., Correa R., Dutt R., Mosunjac M., Oprea-Ilies G., Smith G. (2023). The EMory BrEast Imaging Dataset (EMBED): A Racially Diverse, Granular Dataset of 3.4 Million Screening and Diagnostic Mammographic Images. Radiol. Artif. Intell..

[22] Halling-Brown M.D., Warren L.M., Ward D., Lewis E., Mackenzie A., Wallis M.G., Wilkinson L.S., Given-Wilson R.M., McAvinchey R., Young K.C. (2021). OPTIMAM Mammography Image Database: A Large-Scale Resource of Mammography Images and Clinical Data. Radiol. Artif. Intell..

[23] Lee R.S., Gimenez F., Hoogi A., Miyake K.K., Gorovoy M., Rubin D.L. (2017). A Curated Mammography Data Set for Use in Computer-Aided Detection and Diagnosis Research. Sci. Data.

[24] Strand F. (2022). CSAW-CC (Mammography)—A Dataset for AI Research to Improve Screening, Diagnostics and Prognostics of Breast Cancer.

[25] Kashiwada Y., Takaya E., Hiroya M., Matsuda N., Yashima T., Kobayashi T., Tamiya G., Ueda T. (2025). TOMPEI-CMMD Dataset (Version 1) [Dataset]. The Cancer Imaging Archive. https://www.cancerimagingarchive.net/analysis-result/tompei-cmmd/.

[26] Sawyer-Lee R., Gimenez F., Hoogi A., Rubin D. (2016). Curated Breast Imaging Subset of Digital Database for Screening Mammography (CBIS-DDSM). https://www.cancerimagingarchive.net/collection/cbis-ddsm/.

[27] Tsochatzidis L., Koutla P., Costaridou L., Pratikakis I. (2021). Integrating Segmentation Information into CNN for Breast Cancer Diagnosis of Mammographic Masses. Comput. Methods Programs Biomed..

[28] Ruchay A.N., Kober V.I., Dorofeev K.A., Karnaukhov V.N., Mozerov M.G. (2022). Segmentation of Breast Masses in Digital Mammography Based on U-Net Deep Convolutional Neural Networks. J. Commun. Technol. Electron..

[29] Baccouche A., Garcia-Zapirain B., Castillo Olea C., Elmaghraby A.S. (2021). Connected-UNets: A Deep Learning Architecture for Breast Mass Segmentation. npj Breast Cancer.

[30] Li H., Chen D., Nailon W.H., Davies M.E., Laurenson D.I. (2022). Dual Convolutional Neural Networks for Breast Mass Segmentation and Diagnosis in Mammography. IEEE Trans. Med. Imaging.

[31] Wang R., Ma Y., Sun W., Guo Y., Wang W., Qi Y., Gong X. (2019). Multi-level nested pyramid network for mass segmentation in mammograms. Neurocomputing.

[32] Zhu W., Xiang X., Tran T.D., Hager G.D., Xie X. Adversarial Deep Structured Nets for Mass Segmentation from Mammograms. Proceedings of the 2018 IEEE 15th International Symposium on Biomedical Imaging (ISBI 2018).

[33] Li H., Chen D., Nailon W.H., Davies M.E., Laurenson D. (2018). Improved breast mass segmentation in mammograms with conditional residual U-net. Image Analysis for Moving Organ, Breast, and Thoracic Images.

[34] Zambrano C., Pérez-Pérez N., Coimbra M., Baldeon-Calisto M., Flores-Moyano R., Mora J.R., Camacho O., Benítez D. (2026). MassSeg-Framework: A Breast Mass Detection and Segmentation Framework Based on Deep Learning and an Active Contour Model. Life.

[35] Min H., Wilson D., Huang Y., Liu S., Crozier S., Bradley A.P., Chandra S.S. Fully Automatic Computer-Aided Mass Detection and Segmentation via Pseudo-Color Mammograms and Mask R-CNN. Proceedings of the IEEE 17th International Symposium on Biomedical Imaging (ISBI 2020).

[36] He K., Gkioxari G., Dollar P., Girshick R. Mask R-CNN. Proceedings of the 2017 IEEE International Conference on Computer Vision (ICCV).

[37] Cui C., Li L., Cai H., Fan Z., Zhang L., Dan T., Li J., Wang J. (2021). The Chinese Mammography Database (CMMD): An Online Mammography Database with Biopsy Confirmed Types for Machine Diagnosis of Breast. https://www.cancerimagingarchive.net/collection/cmmd/.

[38] Devadas P., Mathivanan G. (2026). Mask—Region-Based Convolutional Neural Networks (R-CNN) with Radiomics Integration and Gray Level Co-Occurrence Matrix (GLCM) for Brain Tumor Detection and Segmentation. PLoS ONE.

[39] Aykaç E., Göksungur G., Albayrak G.S., Yüksel M.E. (2025). A Mask R-CNN-Based Approach for Brain Aneurysm Detection and Segmentation from TOF-MRA Data. Brain Sci..

[40] Felfeliyan B., Hareendranathan A., Kuntze G., Jaremko J.L., Ronsky J.L. (2022). Improved-Mask R-CNN: Towards an Accurate Generic MSK MRI Instance Segmentation Platform (Data from the Osteoarthritis Initiative). Comput. Med. Imaging Graph..

[41] Yuan J. (2024). Brain Tumor Image Segmentation Method Using Hybrid Attention Module and Improved Mask RCNN. Sci. Rep..

[42] Zhang Y., Chan S., Park V.Y., Chang K.-T., Mehta S., Kim M.J., Combs F.J., Chang P., Chow D., Parajuli R. (2022). Automatic Detection and Segmentation of Breast Cancer on MRI Using Mask R-CNN Trained on Non–Fat-Sat Images and Tested on Fat-Sat Images. Acad. Radiol..

[43] Sahin M.E., Ulutas H., Yuce E., Erkoc M.F. (2023). Detection and Classification of COVID-19 by Using Faster R-CNN and Mask R-CNN on CT Images. Neural Comput. Appl..

[44] Chen X., Wei X., Tang M., Liu A., Lai C., Zhu Y., He W. (2021). Liver Segmentation in CT Imaging with Enhanced Mask Region-Based Convolutional Neural Networks. Ann. Transl. Med..

[45] Chiwhane S., Shrotriya L., Dhumane A., Kothari S., Dharrao D., Bagane P. (2024). Data Mining Approaches to Pneumothorax Detection: Integrating Mask-RCNN and Medical Transfer Learning Techniques. MethodsX.

[46] Ahmed L., Iqbal M.M., Aldabbas H., Khalid S., Saleem Y., Saeed S. (2023). Images Data Practices for Semantic Segmentation of Breast Cancer Using Deep Neural Network. J. Ambient Intell. Humaniz. Comput..

[47] Jahangeer G.S.B., Thambidurai D.R. (2022). Detecting Breast Cancer Using Novel Mask R-CNN Techniques. Expert Syst..

[48] Bhatti H.M.A., Li J., Siddeeq S., Rehman A., Manzoor A. Multi-Detection and Segmentation of Breast Lesions Based on Mask RCNN-FPN. Proceedings of the 2020 IEEE International Conference on Bioinformatics and Biomedicine (BIBM).

[49] Beuque M.P.L., Lobbes M.B.I., Van Wijk Y., Widaatalla Y., Primakov S., Majer M., Balleyguier C., Woodruff H.C., Lambin P. (2023). Combining Deep Learning and Handcrafted Radiomics for Classification of Suspicious Lesions on Contrast-Enhanced Mammograms. Radiology.

[50] Fan M., Zheng H., Zheng S., You C., Gu Y., Gao X., Peng W., Li L. (2020). Mass Detection and Segmentation in Digital Breast Tomosynthesis Using 3D-Mask Region-Based Convolutional Neural Network: A Comparative Analysis. Front. Mol. Biosci..

[51] Otsu N. (1979). A Threshold Selection Method from Gray-Level Histograms. IEEE Trans. Syst. Man. Cybern..

[52] Xu C., Prince J.L. (1998). Snakes, Shapes, and Gradient Vector Flow. IEEE Trans. Image Process..

[53] Chan T.F., Vese L.A. (2001). Active Contours without Edges. IEEE Trans. Image Process..

[54] Wang C., Pedrycz W., Li Z., Zhou M. (2021). Residual-driven Fuzzy C-Means Clustering for Image Segmentation. IEEE/CAA J. Autom. Sin..

[55] Ma J., He Y., Li F., Han L., You C., Wang B. (2024). Segment anything in medical images. Nat. Commun..

[56] Rosset A., Spadola L., Ratib O. (2004). OsiriX: An Open-Source Software for Navigating in Multidimensional DICOM Images. J. Digit. Imaging.

[57] Ren S., He K., Girshick R., Sun J. (2017). Faster R-CNN: Towards Real-Time Object Detection with Region Proposal Networks. IEEE Trans. Pattern Anal. Mach. Intell..

[58] Lin T.-Y., Dollar P., Girshick R., He K., Hariharan B., Belongie S. Feature Pyramid Networks for Object Detection. Proceedings of the 2017 IEEE Conference on Computer Vision and Pattern Recognition (CVPR).

[59] Deng J., Dong W., Socher R., Li L.-J., Li K., Fei-Fei L. ImageNet: A Large-Scale Hierarchical Image Database. Proceedings of the 2009 IEEE Conference on Computer Vision and Pattern Recognition.

[60] Tian J., Jin Q., Wang Y., Yang J., Zhang S., Sun D. (2024). Performance Analysis of Deep Learning-Based Object Detection Algorithms on COCO Benchmark: A Comparative Study. J. Eng. Appl. Sci..

[61] Chen W., Su L., Lin Z., Chen X., Li T. (2023). Instance Segmentation of Irregular Deformable Objects for Power Operation Monitoring Based on Multi-Instance Relation Weighting Module. Electronics.

[62] Kass M., Witkin A., Terzopoulos D. (1988). Snakes: Active Contour Models. Int. J. Comput. Vis..

[63] Hemalatha R.J., Thamizhvani T.R., Dhivya A.J.A., Joseph J.E., Babu B., Chandrasekaran R., Koprowski R. (2018). Active Contour Based Segmentation Techniques for Medical Image Analysis. Medical and Biological Image Analysis.

[64] Müller D., Soto-Rey I., Kramer F. (2022). Towards a Guideline for Evaluation Metrics in Medical Image Segmentation. BMC Res. Notes.

[65] Jafrasteh B., Lubián-Gutiérrez M., Lubián-López S.P., Benavente-Fernández I. (2024). Enhanced Spatial Fuzzy C-Means Algorithm for Brain Tissue Segmentation in T1 Images. Neuroinformatics.

[66] Choudhry M.S., Kapoor R. (2016). Performance Analysis of Fuzzy C-Means Clustering Methods for MRI Image Segmentation. Procedia Comput. Sci..

[67] Bhalla K., Dahiya S., Gosain A. (2025). Kernelized Type 2 Intuitionistic Fuzzy C Means: A robust approach for brain MRI image segmentation. Discov. Comput..

[68] Kirillov A., Mintun E., Ravi N., Mao H., Rolland C., Gustafson L., Xiao T., Whitehead S., Berg A.C., Lo W.-Y. (2023). Segment Anything. arXiv.

[69] Yeghiazaryan V., Voiculescu I. (2018). Family of Boundary Overlap Metrics for the Evaluation of Medical Image Segmentation. J. Med. Imaging.

[70] Nikolov S., Blackwell S., Zverovitch A., Mendes R., Livne M., De Fauw J., Patel Y., Meyer C., Askham H., Romera-Paredes B. (2021). Clinically Applicable Segmentation of Head and Neck Anatomy for Radiotherapy: Deep Learning Algorithm Development and Validation Study. J. Med. Internet Res..

[71] Badal A., Sharma D., Graff C.G., Zeng R., Badano A. (2021). Mammography and Breast Tomosynthesis Simulator for Virtual Clinical Trials. Comput. Phys. Commun..

[72] U.S. Food and Drug Administration VICTRE: In Silico Breast Imaging Pipeline (RST24MX01.01). https://cdrh-rst.fda.gov/victre-silico-breast-imaging-pipeline.

[73] Oria M., Ferrero R., Andreis C., Vicentini M., Van Engen R., Roozemond C., Lamberti P., Remogna S., Manzin A. (2025). Image-Based Reconstruction of Anthropomorphic Breast Phantoms for Synthetic Mammogram Generation. Comput. Biol. Med..

[74] de Sisternes L., Brankov J.G., Zysk A.M., Schmidt R.A., Nishikawa R.M., Wernick M.N. (2015). A computational model to generate simulated three-dimensional breast masses. Med. Phys..

[75] Maas S.A., Ellis B.J., Ateshian G.A., Weiss J.A. (2012). FEBio: Finite elements for biomechanics. J. Biomech. Eng..

[76] Raina E., Oria M., Salvi M., Manzin A. (2026). A Neural Network Model for the Accurate Detection of Cancer Lesions in Mammography. Phys. Medica.

[77] Wang H., Wang Z., Du M., Yang F., Zhang Z., Ding S., Mardziel P., Hu X. Score-CAM: Score-weighted visual explanations for convolutional neural networks. Proceedings of the 2020 IEEE/CVF Conference on Computer Vision and Pattern Recognition Workshops (CVPRW).

[78] Badano A., Graff C.G., Badal A., Sharma D., Zeng R., Samuelson F.W., Glick S.J., Myers K.J. (2018). Evaluation of Digital Breast Tomosynthesis as Replacement of Full-Field Digital Mammography Using an in Silico Imaging Trial. JAMA Netw. Open.

