# Supplementary material for: Automated Segmentation of Mass Lesions in Mammography: Mask R-CNN Versus Classical Methods, Fuzzy Clustering and MedSAM
Source: Diagnostics (Basel). 2026 Oct 10;16(20):3289. doi: 10.3390/diagnostics16203289 (PMC13702960; doi:10.3390/diagnostics16203289)
Supplement: Supplementary file 1 [file diagnostics-16-03289-s001.zip › diagnostics-4505740-supplementary.pdf]

Supplementary Materials

Automated Segmentation of Mass Lesions in Mammography: Mask R-CNN versus Classical Methods, Fuzzy Clustering and MedSAM

Marta Vicentini [1], Martina Oria [1,2], Eva Raina [1,2], Manuela Durando [3] and Alessandra Manzin [1]

[1] Department of Advanced Materials Metrology and Life Sciences, Istituto Nazionale di Ricerca Metrologica (INRiM), 10135 Torino, Italy

[2] Department of Electronics and Telecommunications, Politecnico di Torino, 10129 Torino, Italy

[3] S.C. RADIOLOGIA 1U - Department of Diagnostic Imaging and Interventional Radiology, A.O.U. Città della Salute e della Scienza di Torino, 10126 Torino, Italy

Corresponding authors: Marta Vicentini (m.vicentini@inrim.it); Alessandra Manzin (a.manzin@inrim.it)

Table S1. Specifications of Mask R-CNN hyperparameters and architecture features corresponding to the optimal configuration (Model 3), as determined in Sub-section 2.3.

Hyperparameter/ Feature	Specification
model.transform.min_size	800 px
model.transform.max_size	1333 px
model.transform.image_mean	[0.485, 0.456, 0.406]
model.transform.image_std	[0.229, 0.224, 0.225]
Architecture	ResNet-50 + FPN
Initial weights	COCO-pretrained
Trainable backbone layers	3
model.rpn.anchor_generator.sizes	32, 64, 128, 256, 512
model.rpn.anchor_generator.aspect_ratios	0.5, 1.0, 2.0
model.rpn._pre_nms_top_n (proposals kept before NMS, train / test)	2000 / 1000
model.rpn._post_nms_top_n (proposals kept after NMS, train / test)	2000 / 1000
model.rpn.nms_thresh (IoU threshold for proposal NMS)	0.7
model.rpn.proposal_matcher.high_threshold (foreground IoU threshold)	0.7
model.rpn.proposal_matcher.low_threshold (background IoU threshold)	0.3
model.rpn.fg_bg_sampler.batch_size_per_image	256
model.rpn.fg_bg_sampler.positive_fraction	0.5
model.rpn.score_thresh (minimum objectness score for proposals)	0.0
model.roi_heads.box_roi_pool.output_size (RoIAlign output resolution)	7 × 7
model.roi_heads.box_roi_pool.sampling_ratio	2
model.roi_heads.nms_thresh (IoU threshold for box NMS)	0.5
model.roi_heads.detections_per_img (max detections kept per image)	100
model.roi_heads.proposal_matcher.high_threshold (foreground IoU threshold)	0.5
model.roi_heads.proposal_matcher.low_threshold (background IoU threshold)	0.5
model.roi_heads.fg_bg_sampler.batch_size_per_image	512
model.roi_heads.fg_bg_sampler.positive_fraction	0.25
model.roi_heads.box_coder.weights (bounding-box regression weights)	(10.0, 10.0, 5.0, 5.0)
model.roi_heads.mask_roi_pool.output_size	14 × 14
model.roi_heads.mask_roi_pool.sampling_ratio	2
model.roi_heads.mask_predictor (hidden layer dimension)	256
model.roi_heads.box_predictor.cls_score.out_features (number of classes, including background)	2

Training Hyperparameter/ Feature	Specification
Optimizer	SGD
Learning rate	0.005
Momentum	0.9
Weight decay	5×10^{-4}
LR scheduler	StepLR
LR step size	5 epochs
LR decay factor (γ)	0.1
Number of epochs	25
Batch size (train / validation)	2 / 1
Data augmentation	None
Mixed precision (AMP)	Disabled
Backbone trainable layers	3

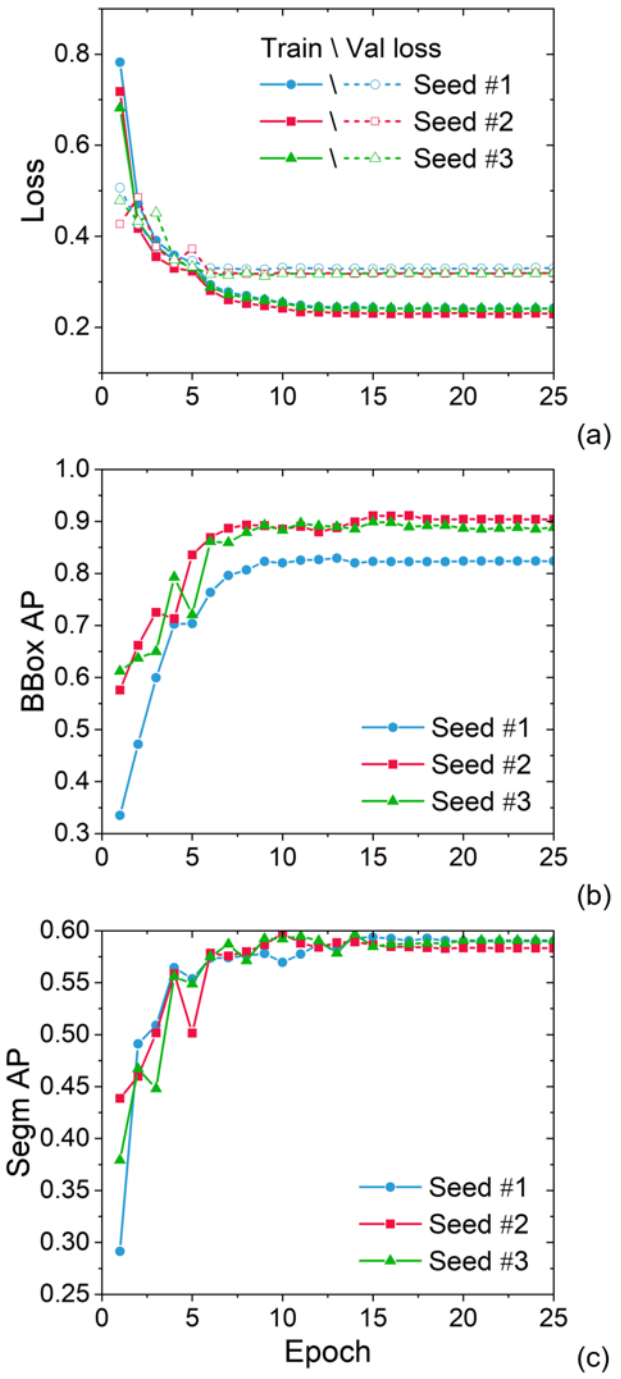

Figure S1. Comparison of optimal configuration of Mask R-CNN (Model 3) for three seed values, in terms of **(a)** training and validation losses, **(b)** bounding box average precision (BBox AP) and **(c)** segmentation average precision (Segm AP). For BBox AP and Segm AP values, we consider IoU = 0.5:0.95.

Table S2. List of the applied image pre-processing pipelines (relevant for Otsu thresholding, CVS and FCM methods).

ID	Pipeline	Details
1	No transformation applied	None
2	Normalization (min max rescaling)	Output range 0 to 1
3	Normalization followed by morphological opening	Disk shaped structuring element, radius 2 pixels
4	Normalization, morphological opening, contrast adjustment	Disk element with radius of 2 pixels
5	Normalization followed by Gaussian filtering	Standard deviation 2.5
6	Normalization, Gaussian filtering, contrast adjustment	Standard deviation 2.5
7	Normalization followed by contrast adjustment	Input range 0.3 to 0.7
8	Normalization, contrast adjustment, morphological opening	Disk element with radius of 2 pixels
9	Normalization, contrast adjustment, morphological closing	Disk element with radius of 2 pixels
10	Normalization, contrast adjustment, morphological opening	Disk element with radius of 3 pixels
11	Normalization, contrast adjustment, morphological opening, morphological closing	Opening with disk element of 3 pixels radius; closing with disk element of 2 pixels radius
12	Principal component analysis	10 components retained
13	Principal component analysis	20 components retained
14	Principal component analysis	50 components retained
15	Principal component analysis	75 components retained
16	Principal component analysis followed by morphological closing	10 components; radius 4 pixels
17	Principal component analysis, morphological closing, morphological opening	5 components; closing disk element of 4 pixels radius; opening with disk element of 2 pixels radius
18	Normalization followed by contrast limited adaptive histogram equalization	Clip limit 0.01, uniform distribution
19	Normalization, top hat morphological filtering, rescaling	Disk element with radius of 12 pixels; output range 0 to 1
20	Normalization, bottom hat morphological filtering, rescaling	Radius 12 pixels; output range 0 to 1
21	Normalization followed by anisotropic diffusion filtering	10 iterations, gradient threshold 10
22	Normalization followed by wavelet-based denoising	\
23	Normalization, adaptive histogram equalization, median filtering	kernel with size of 3 × 3 pixels
24	Normalization followed by intensity quantization	\
25	Normalization, intensity quantization, morphological opening	\
26	Normalization, intensity quantization, morphological closing	\

Radius values refer to disk shaped structuring elements.

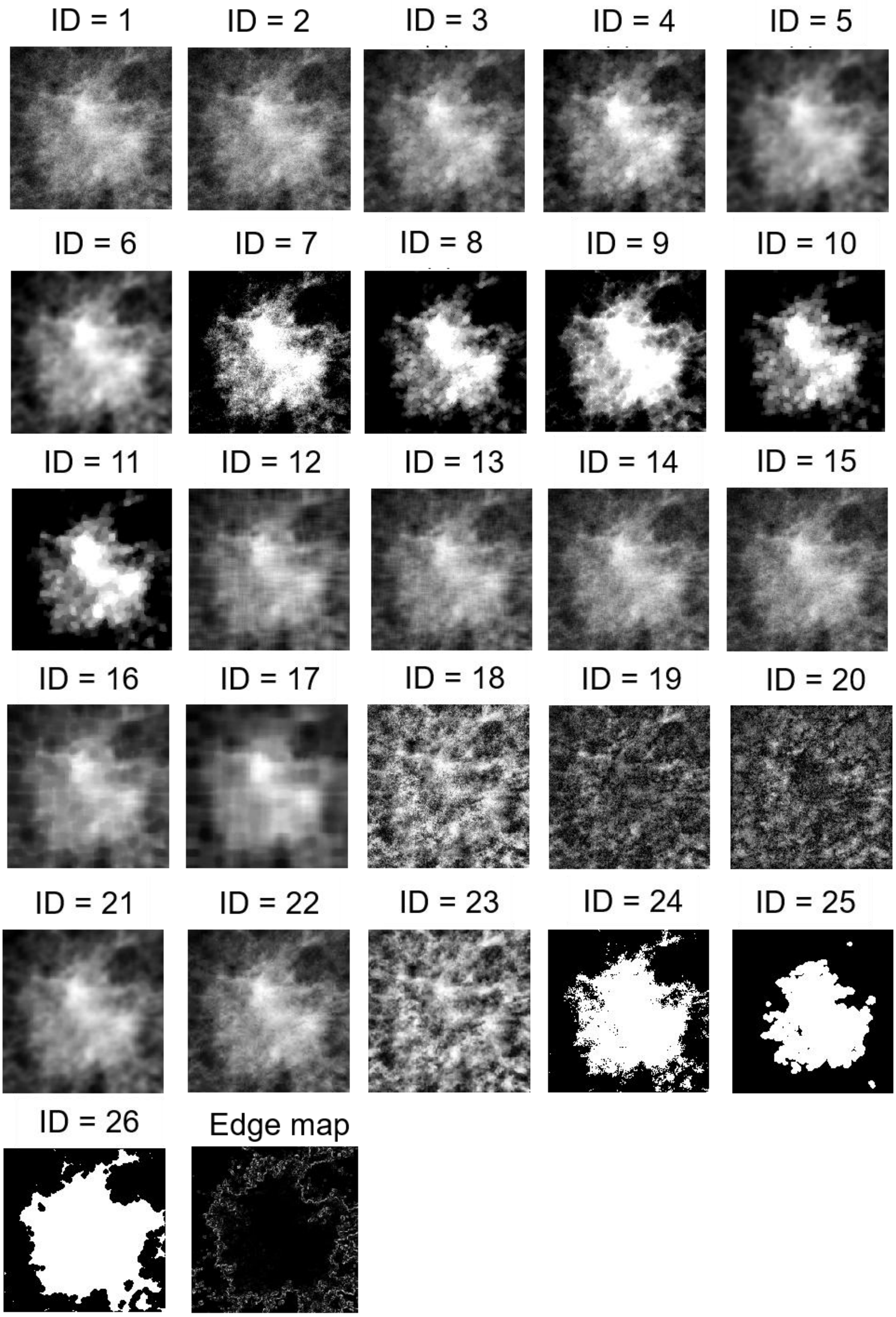

Figure S2. Examples of application of the pre-processing pipelines listed in Table SII to an image from CSAW-CC database. The original patch extracted from the real mammogram corresponds to ID 1. The last patch reports the corresponding edge map used for the application of the GVF active contour method.

Table S3. Application of the Wilcoxon rank sum test on the segmentation metrics obtained from the test on the 112 images from CSAW-CC and 103 images from TOMPEI-CMMD, reported in Sub-section 3.2. For each metric, the test statistic (rank sum), the normalized statistic (Z), and the corresponding p-value are reported.

Metric	Rank sum	Z	p-value
IoU	12462	0.8032	0.4219
DSC	12462	0.8032	0.4219
Precision	11887	-0.4576	0.6473
Recall	12676	1.2717	0.2035
Accuracy	11731	-0.7999	0.4238
HD95	12136	0.0878	0.9301
ASSD	11982	-0.2491	0.8033
NSD05	13716	3.5529	3.8101E-4*
NSD075	14041	4.2672	1.9791E-5*
NSD1	14314	4.8669	1.1337E-6*
NSD2	14266	5.0276	4.9669E-7*

Null hypothesis: median1 = median2.

The * symbol indicates statistically significant differences ($p < 0.05$) in metric distribution between the two databases.

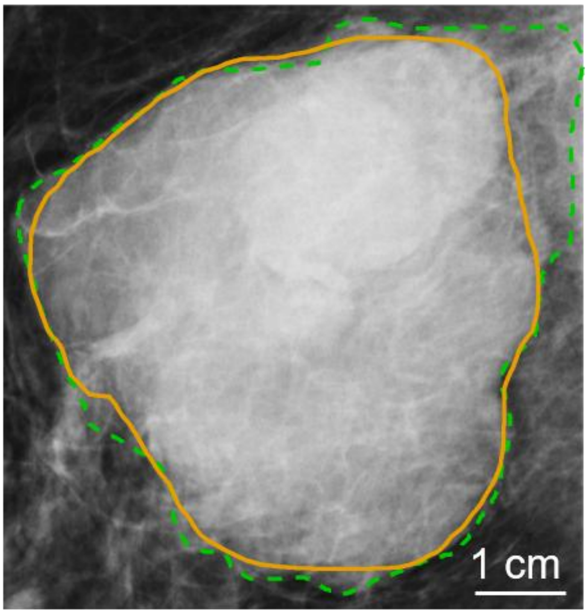

Figure S3. Visual comparison between the segmentation generated by the optimal configuration of Mask R-CNN (Model 3), highlighted by the orange curve, and the reference mask boundary (green dashed curve) for a mass lesion from the CSAW-CC dataset. This specific case reaches the highest value of the 95th percentile of the Hausdorff distance (HD95) across the entire test set, with a value of 110 px (corresponding to approximately 7.7 mm). Further details about source mammograms can be found in Table SVIII.

Table S4. Segmentation metric scores obtained with the optimal configuration of Mask R-CNN (Model 3), when applied to the images in Figure 6. The notation Fig. 6x I–IV identifies the position of the images within panel x, where x = a, b, c, ordered from left to right (from the first column, I, to the fourth column, IV).

Image	IoU	DSC	Precision	Recall	Accuracy	HD95	ASSD	NSD05	NSD075	NSD1	NSD2
Fig.6a-I	0.89	0.94	0.94	0.93	0.95	10.35	4.90	0.85	0.98	1	1
Fig.6a-II	0.90	0.95	0.94	0.95	0.96	9.85	3.99	0.94	0.98	1	1
Fig.6a-III	0.91	0.95	0.98	0.92	0.96	10.11	4.74	0.79	0.93	0.95	1
Fig.6a-IV	0.92	0.96	0.99	0.93	0.97	7.07	3.92	0.92	0.99	1	1
Fig.6b-I	0.78	0.87	0.79	0.98	0.91	13.18	6.67	0.64	0.86	0.98	1
Fig.6b-II	0.76	0.86	0.78	0.96	0.90	19.65	7.39	0.62	0.74	0.85	1
Fig.6b-III	0.80	0.89	0.96	0.82	0.92	15.68	8.32	0.40	0.53	0.72	1
Fig.6b-IV	0.80	0.89	0.97	0.82	0.94	14.69	5.56	0.65	0.73	0.81	0.95
Fig.6c-I	0.53	0.69	0.56	0.91	0.85	13.01	7.74	0.51	0.73	0.94	1
Fig.6c-II	0.55	0.71	0.57	0.94	0.86	12.53	6.00	0.63	0.82	0.99	1
Fig.6c-III	0.47	0.64	0.47	1.00	0.84	14.38	8.43	0.35	0.76	0.97	1
Fig.6c-IV	0.53	0.70	0.57	0.90	0.90	14.97	6.39	0.60	0.76	0.91	1

Metric abbreviations: IoU = intersection over union; DSC = Dice similarity coefficient; HD95 = 95th percentile of the Hausdorff distance; ASSD = average symmetric surface distance; NSD05 = normalized surface distance with tolerance distance τ of 0.5 mm; NSD075 = normalized surface distance with τ = 0.75 mm; NSD1 = normalized surface distance with τ = 1 mm; NSD2 = normalized surface distance with τ = 2 mm.

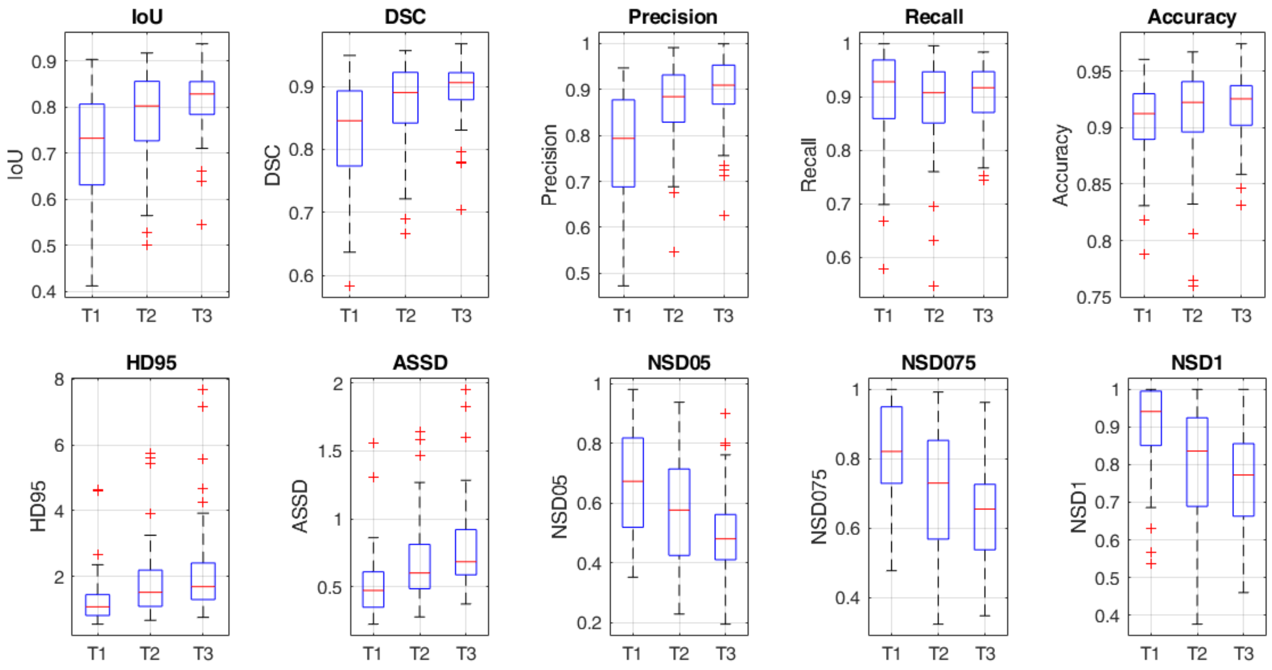

Figure S4. Box plots of metric distributions evaluated using the optimal configuration of Mask R-CNN (Model 3), categorized into tertiles based on the annotated lesion equivalent diameter. T1: diameter lower than 10 mm; T2: diameter between 10 mm and 15 mm; T3: diameter higher than 15 mm. The results refer to the test on the 112 images from CSAW-CC and 103 images from TOMPEI-CMMD, reported in Sub-section 3.2.

Table S5. Correlation analysis between segmentation metrics and lesion size for the results obtained by using the optimal configuration of Mask R-CNN (Model 3) on the 112 images from CSAW-CC and 103 images from TOMPEI-CMMD, as reported in Sub-section 3.2. For each metric, the Spearman correlation coefficient (ϱ) and the corresponding p-value are listed, calculated by comparing metric values against lesion diameter, along with the Kruskal-Wallis test statistic (H) and the corresponding p-value, calculated by comparing metric distributions across lesion size [illegible]

**Metric**	**Spearman (ϱ)**	**Spearman (p-value)**	**Kruskal-Wallis (H)**	**Kruskal-Wallis (p-value)**
IoU	0.40623	5.9878e-10	32.992	6.8538e-08
DSC	0.40623	5.9878e-10	32.992	6.8538e-08
Precision	0.4484	4.9578e-12	43.126	4.3177e-10
Recall	-0.096684	0.15774	4.1339	0.12657
Accuracy	0.12258	0.072865	5.2801	0.071359
HD95	0.46699	4.817e-13	39.038	3.334e-09
ASSD	0.48734	< 1e-16	42.144	7.0574e-10
NSD05	-0.44832	5.0058e-12	32.776	7.6343e-08
NSD075	-0.49596	9.4871e-15	42.145	7.0531e-10
NSD1	-0.50919	1.3901e-15	44.45	2.2274e-10

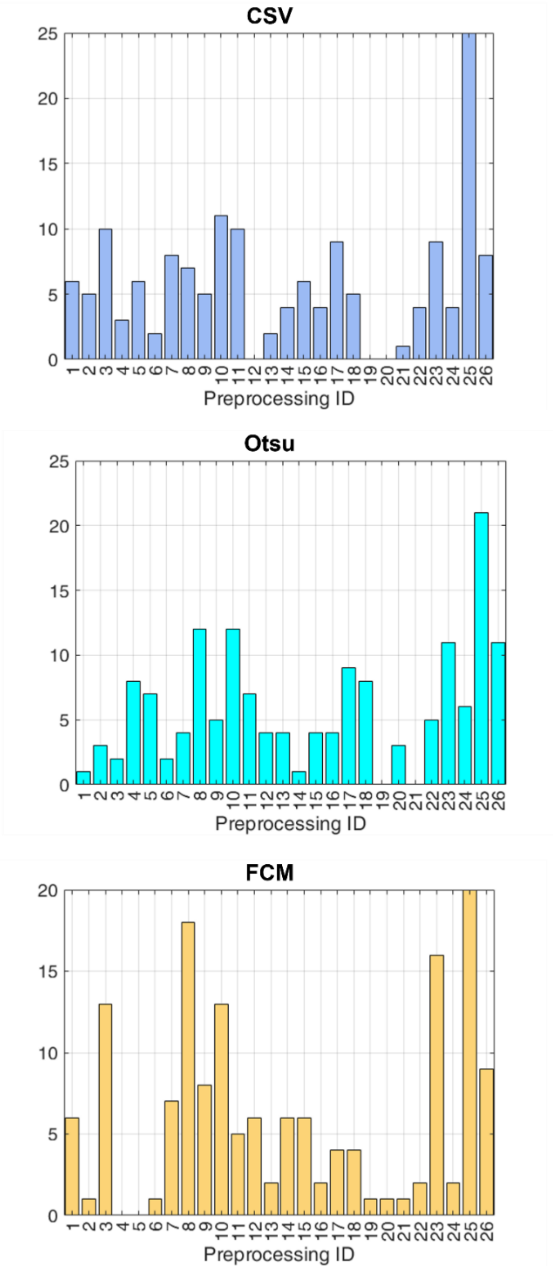

Figure S5. Histograms showing the number of image patches for which each pre-processing technique leads to superior performance, based on the average of overlap-based metrics (relevant for the test analysis reported in Sub-section 3.3). Results for the CVS method are reported at the top, Otsu thresholding in the middle, and FCM at the bottom.

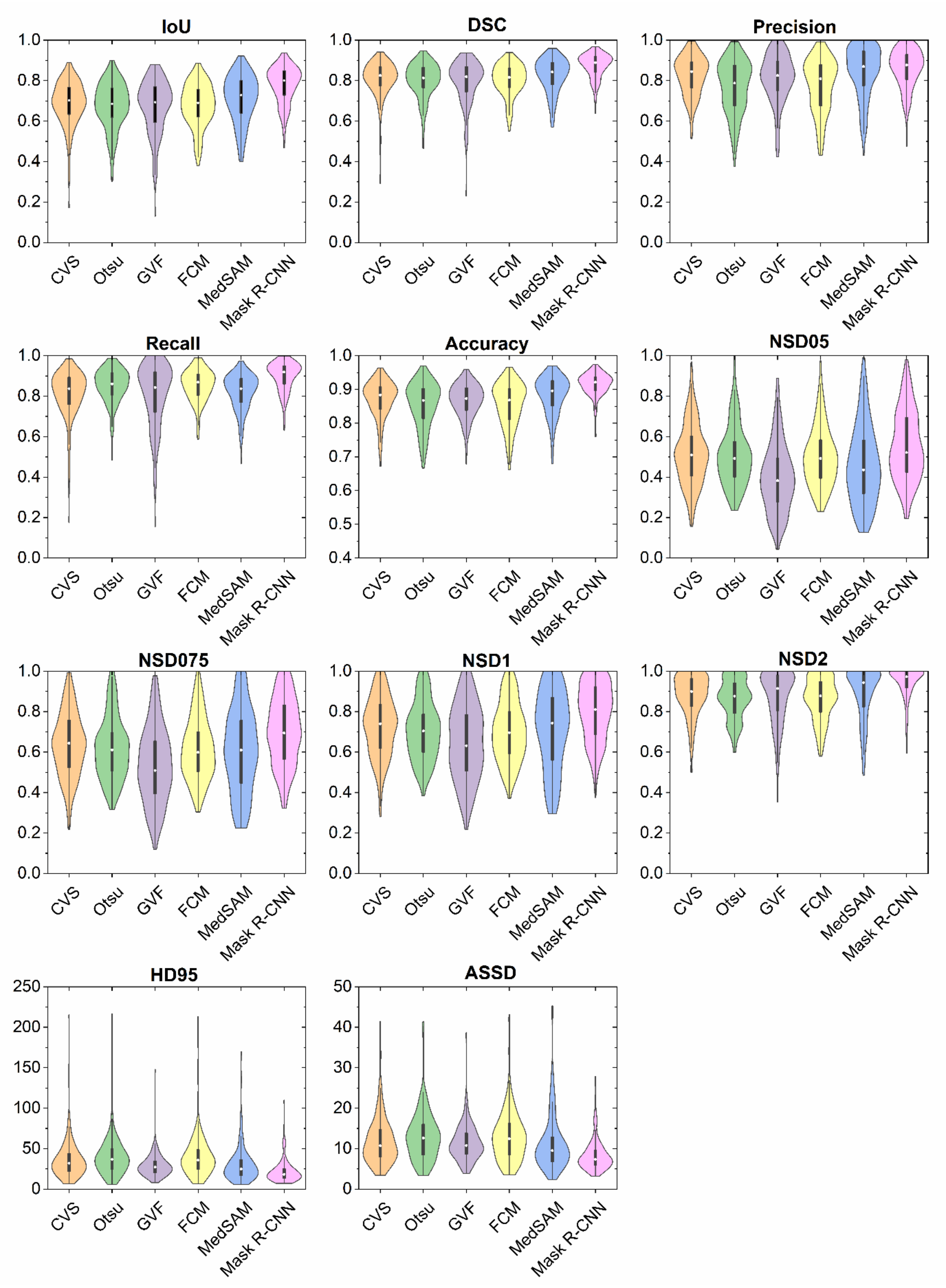

Figure S6. Violin plots of metric distribution comparing Mask R-CNN model with other segmentation methods (CVS, Otsu thresholding, GVF active contour, FCM and MedSAM). The performance is evaluated by considering overlap-based metrics (IoU, DSC, Precision, Recall and Accuracy), NSD with τ equal to 0.5 mm, 0.75 mm, 1 mm and 2 mm, HD95 (values in pixels) and ASSD (values in pixels). The results refer to the test analysis reported in Sub-section 3.3.

Table S6. Segmentation metric scores obtained for the images in Figure 8, by applying the optimal configuration of Mask R-CNN (Model 3), CVS, Otsu thresholding, GVF active contour, FCM, and MedSAM. The label indicates the corresponding column in Figure 8, while the segmentation type refers to the row.

Label	Segmentation	IoU	DSC	Precision	Recall	Accuracy	HD95	ASSD	NSD05	NSD075	NSD1	NSD2
a	Mask R-CNN	0.86	0.92	0.90	0.95	0.94	13.00	5.30	0.73	0.90	0.99	1.00
a	CVS	0.79	0.88	0.91	0.86	0.91	18.44	6.61	0.66	0.79	0.89	0.99
a	Otsu	0.80	0.89	0.90	0.87	0.91	16.28	6.48	0.67	0.79	0.91	0.99
a	GVF	0.80	0.89	0.85	0.94	0.91	17.00	7.38	0.54	0.72	0.91	1.00
a	FCM	0.79	0.88	0.9	0.87	0.91	16.6	6.81	0.65	0.77	0.9	0.99
a	MedSAM	0.75	0.85	0.96	0.77	0.9	28.3	9.03	0.46	0.72	0.87	0.95
b	Mask R-CNN	0.78	0.87	0.79	0.98	0.91	13.18	6.67	0.64	0.86	0.98	1.00
b	CVS	0.84	0.91	0.91	0.92	0.94	9.85	3.44	0.85	0.98	1.00	1.00
b	Otsu	0.83	0.91	0.89	0.93	0.94	9.89	3.74	0.83	0.97	1.00	1.00
b	GVF	0.85	0.92	0.89	0.95	0.95	9.31	3.86	0.83	0.98	1.00	1.00
b	FCM	0.83	0.91	0.9	0.92	0.94	9.22	3.55	0.83	0.98	1	1
b	MedSAM	0.83	0.91	0.91	0.91	0.94	12.4	4.25	0.87	0.92	0.97	1
c	Mask R-CNN	0.76	0.86	0.78	0.96	0.90	19.65	7.39	0.62	0.74	0.85	1.00
c	CVS	0.57	0.73	0.65	0.84	0.79	38.60	13.29	0.44	0.56	0.63	0.87
c	Otsu	0.34	0.51	0.54	0.48	0.68	36.80	12.71	0.44	0.54	0.65	0.89
c	GVF	0.69	0.82	0.75	0.90	0.86	26.91	9.19	0.48	0.65	0.77	0.95
c	FCM	0.59	0.74	0.63	0.91	0.78	32.5	12.7	0.41	0.53	0.61	0.91
c	MedSAM	0.62	0.77	0.95	0.64	0.86	22	10.7	0.32	0.51	0.69	1
d	Mask R-CNN	0.85	0.92	0.93	0.91	0.95	13.95	5.48	0.74	0.86	0.91	1.00
d	CVS	0.73	0.85	0.89	0.81	0.90	21.15	8.86	0.52	0.65	0.78	0.98
d	Otsu	0.73	0.84	0.86	0.83	0.90	21.24	8.87	0.53	0.65	0.78	0.98
d	GVF	0.65	0.79	0.70	0.90	0.84	22.74	11.36	0.32	0.51	0.62	0.95
d	FCM	0.72	0.83	0.84	0.83	0.89	20.6	9.14	0.5	0.62	0.75	0.98
d	MedSAM	0.8	0.89	0.9	0.88	0.93	16.3	6.65	0.6	0.76	0.86	1

Metric abbreviations: IoU = intersection over union; DSC = Dice similarity coefficient; HD95 = 95th percentile of the Hausdorff distance; ASSD = average symmetric surface distance; NSD05 = normalized surface distance with tolerance distance τ of 0.5 mm; NSD075 = normalized surface distance with τ = 0.75 mm; NSD1 = normalized surface distance with τ = 1 mm; NSD2 = normalized surface distance with τ = 2 mm.

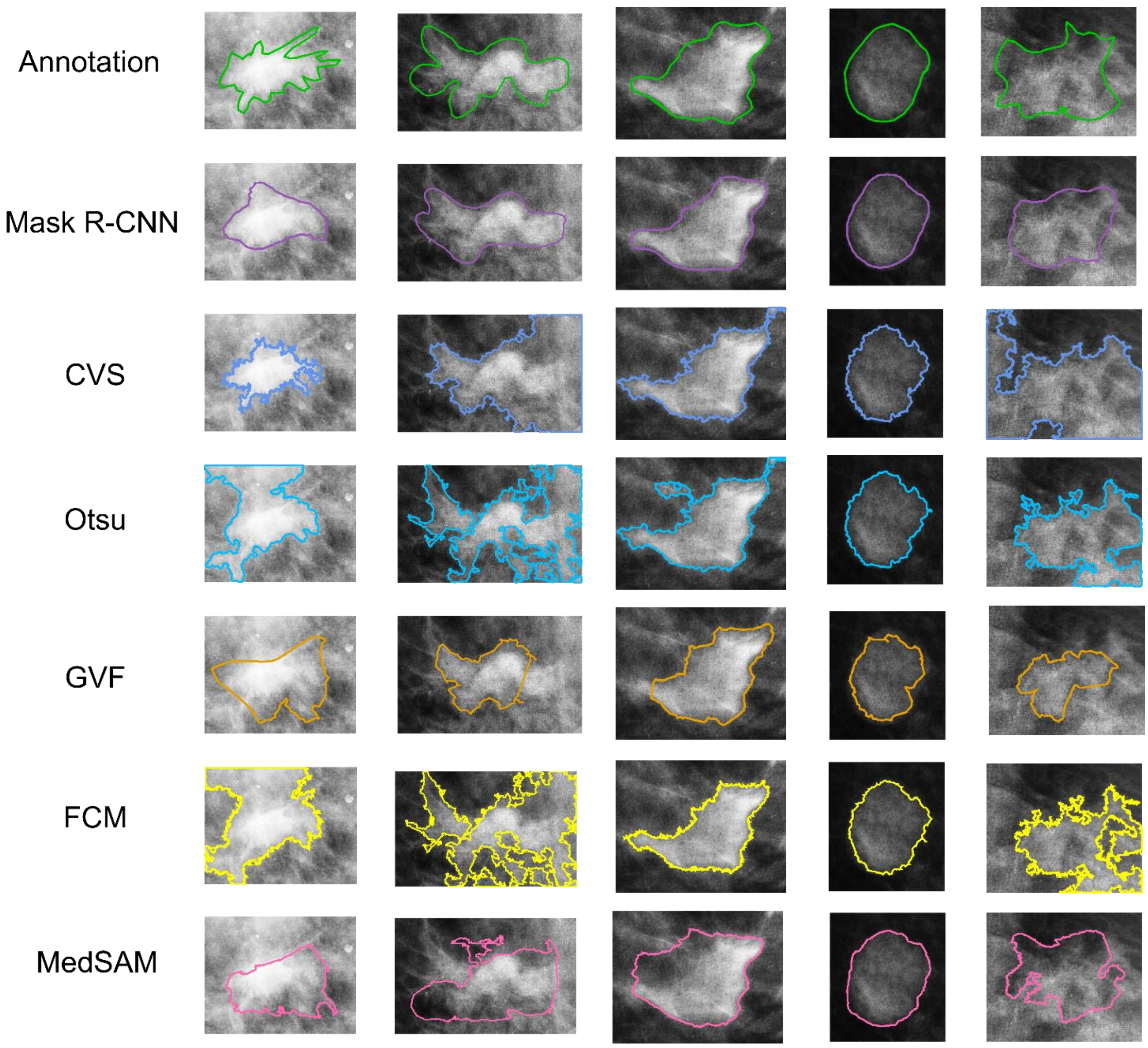

Figure S7. Application of the optimal configuration of Mask R-CNN (Model 3) (purple curves), classical segmentation methods (CVS in light blue, Otsu thresholding in cyan and GVF active contour in orange), FCM (yellow curves), and the SOTA model MedSAM (pink curves) to a set of mass lesions in mammograms from TOMPEI-CMMD database. Green curves depict the corresponding annotations provided by the dataset. Further details about source mammograms can be found in Table SVIII.

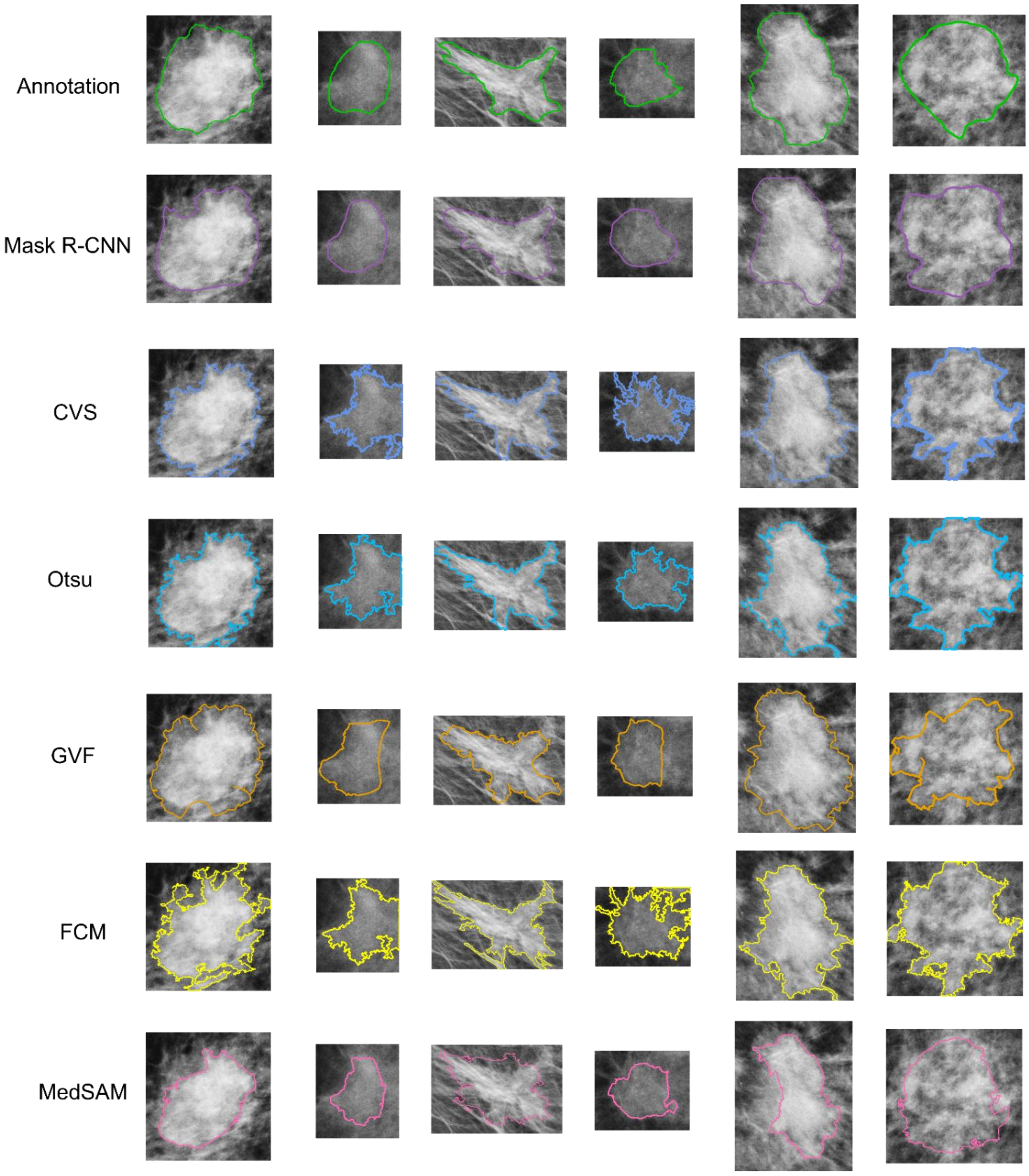

Figure S8. Application of the optimal configuration of Mask R-CNN (Model 3) (purple curves), classical segmentation methods (CVS in light blue, Otsu thresholding in cyan and GVF active contour in orange), FCM (yellow curves), and the SOTA model MedSAM (pink curves) to a set of mass lesions in mammograms from CSAW-CC database. Green curves depict the corresponding annotations provided by the dataset. Further details about source mammograms can be found in Table SVIII.

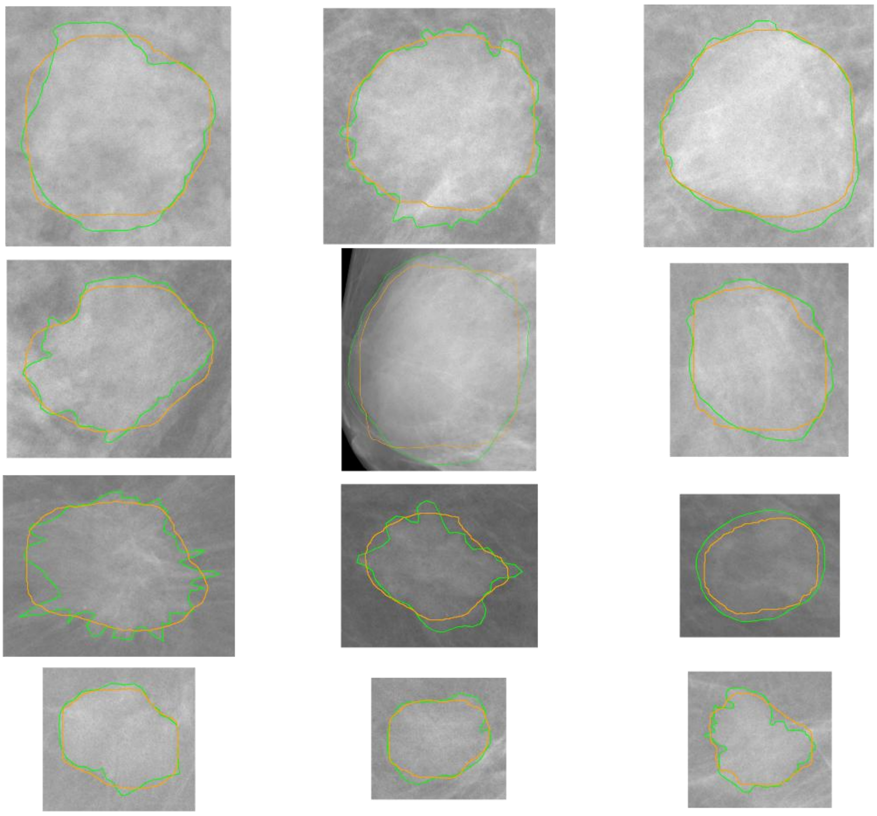

Figure S9. Application of the optimal configuration of Mask R-CNN (Model 3) (orange curves) to a set of mass lesions in mammograms from INbreast database. The segmentation results are compared to the annotations provided by the dataset (green curves). Further details about source mammograms can be found in Table SVIII.

Table S7. Segmentation metric scores obtained by applying the optimal configuration of Mask R-CNN (Model 3) on the 113 images from INbreast database. For each metric, the mean, standard deviation, minimum value, and maximum value are reported.

	**IoU**	**DSC**	**Precision**	**Recall**	**Accuracy**	**HD95**	**ASSD**	**NSD05**	**NSD075**	**NSD1**	**NSD2**
Mean	0.80	0.89	0.90	0.88	0.90	30.25	12.27	0.46	0.60	0.70	0.89
Standard Deviation	0.07	0.05	0.10	0.05	0.03	17.75	6.93	0.20	0.22	0.21	0.13
Minimum	0.57	0.73	0.61	0.74	0.83	5.30	3.01	0.10	0.15	0.21	0.46
Maximum	0.93	0.96	1.00	0.99	0.97	99.78	42.97	1.00	1.00	1.00	1.00

Table S8. Details of the cropped mammograms shown in Figures 6, 8, S4, S7, S8 and S9. Each image is identified by the figure number and its position in the figure (row and column). For each image, the table reports the source database (CSAW-CC, TOMPEI-CMMD or INbreast), the patient/image label, the image details (laterality: L/R = left/right breast, view: CC/MLO = craniocaudal/mediolateral oblique), the lesion details (reported using the terminology of each database), and the coordinates of the bounding box (x_min, x_max, y_min, y_max, in pixels) used to crop the region of interest. For TOMPEI-CMMD, the view information is retrieved from the corresponding annotation files.

Figure	Row	Column	Database	Image identification	Image details	Lesion details	Box coordinates (x_min,x_max,y_min,y_max)
Fig.6	1	1	CSAW-CC	01910	R_MLO	Cancer, invasive	(2679,2881,2274,2480)
Fig.6	1	2	CSAW-CC	04439	R_CC	Cancer, invasive	(2215,2454,2260,2434)
Fig.6	1	3	TOMPEI-CMMD	D1-1375	L_MLO	Malignant, mass	(267,500,1494,1715)
Fig.6	1	4	TOMPEI-CMMD	D1-1723	L_MLO	Malignant, mass	(290,479,412,621)
Fig.6	2	1	CSAW-CC	03809	R_CC	Cancer, invasive	(1967,2129,1915,2126)
Fig.6	2	2	CSAW-CC	04325	R_MLO	Cancer, invasive	(2300,2469,1985,2167)
Fig.6	2	3	TOMPEI-CMMD	D1-1312	L_MLO	Malignant, mass	(228,491,1172,1381)
Fig.6	2	4	TOMPEI-CMMD	D1-1753	L_MLO	Malignant, mass	(11,198,1239,1423)
Fig.6	3	1	CSAW-CC	03809	R_MLO	Cancer, invasive	(1927,2044,2125,2222)
Fig.6	3	2	CSAW-CC	02427	\	Cancer, invasive	(1,81,1633,1744)
Fig.6	3	3	CSAW-CC	01567	L_MLO	Cancer, invasive	(1569,1667,2642,2744)
Fig.6	3	4	TOMPEI-CMMD	D1-1392	R_MLO	Malignant, mass	(1319,1402,1419,1507)
Fig.8	1	1	CSAW-CC	01910	R_CC	Cancer, invasive	(2765,2977,1535,1749)
Fig.8	1	2	CSAW-CC	03809	R_CC	Cancer, invasive	(1967,2129,1915,2126)
Fig.8	1	3	CSAW-CC	04325	R_MLO	Cancer, invasive	(2300,2469,1985,2167)
Fig.8	1	4	TOMPEI-CMMD	D1-1170	R_MLO	Malignant, mass	(1734,1901,1531,1702)
Fig.S4	\	\	CSAW-CC	05045	R_MLO	Cancer	(2360,3328,1320,2329)
Fig.S7	1	1	TOMPEI-CMMD	D1-0391	L_MLO	Malignant, mass	(491,739,1350,1541)
Fig.S7	1	2	TOMPEI-CMMD	D1-1047	L_MLO	Malignant, mass	(142,508,1493,1726)
Fig.S7	1	3	TOMPEI-CMMD	D1-1054	L_MLO	Malignant, mass	(449,727,1429,1645)
Fig.S7	1	4	TOMPEI-CMMD	D1-1723	L_MLO	Malignant, mass	(290,479,412,621)
Fig.S7	1	5	TOMPEI-CMMD	D1-1769	L_MLO	Malignant, mass	(10,264,527,738)
Fig.S8	1	1	CSAW-CC	01433	R_CC	Cancer, invasive	(1886,2233,1173,1529)
Fig.S8	1	2	CSAW-CC	02019	R_MLO	Cancer, invasive	(1552,1736,1345,1554)
Fig.S8	1	3	CSAW-CC	04808	L_MLO	Cancer, invasive	(310,946,2113,2544)
Fig.S8	1	4	CSAW-CC	10427	L_CC	Cancer, invasive	(55,266,1698,1874)
Fig.S8	1	5	CSAW-CC	01310	L_MLO	Cancer, invasive	(227,554,1739,2156)
Fig.S8	1	6	CSAW-CC	08061	R_MLO	Cancer, invasive	(2363,2802,1364,1798)
Fig.S9	1	1	INbreast	22678646	L_CC	Mass, Bi-Rads 3	(1586,1962,72,426)

Fig.S9	1	2	INbreast	50996352	R_MLO	Mass, Bi-Rads 4c	(1351,1721,1840,2203)
Fig.S9	1	3	INbreast	50996406	R_CC	Mass, Bi-Rads 4c	(1174,1554,2188,2548)
Fig.S9	2	1	INbreast	50997515	R_CC	Mass, Bi-Rads 4a	(1158,1468,2909,3261)
Fig.S9	2	2	INbreast	20587928	R_MLO	Mass, Bi-Rads 3	(1262,2077,1308,2021)
Fig.S9	2	3	INbreast	50994354	R_MLO	Mass, Bi-Rads 4a	(800,1103,2208,2488)
Fig.S9	3	1	INbreast	20587664	R_MLO	Mass, Bi-Rads 5	(603,1422,2144,3063)
Fig.S9	3	2	INbreast	22427751	L_MLO	Mass, Bi-Rads 5	(1182,1438,673,981)
Fig.S9	3	3	INbreast	51049107	L_CC	Mass, Bi-Rads 2	(1457,1634,414,570)
Fig.S9	4	1	INbreast	53586896	L_CC	Mass, Bi-Rads 2	(2589,2814,1312,1551)
Fig.S9	4	2	INbreast	53586869	L_MLO	Mass, Bi-Rads 2	(2988,3179,1378,1589)
Fig.S9	4	3	INbreast	53581460	R_CC	Mass, Bi-Rads 6	(814,1028,1880,2105)
